# Role of leisure sedentary behavior on type 2 diabetes and glycemic homeostasis: a Mendelian randomization analysis

**DOI:** 10.3389/fendo.2023.1221228

**Published:** 2023-11-23

**Authors:** Hui Jia, Yifan Liu, Dandan Liu

**Affiliations:** ^1^ Department of Endocrinology, The Eighth Affiliated Hospital of Sun Yat-sen University, Sun Yat-sen University, Shenzhen, China; ^2^ State Key Laboratory of Oncology in South China, Sun Yat-sen University, Guangzhou, China

**Keywords:** type 2 diabetes, Mendelian randomization analysis, glycemic traits, glycemic homeostasis, BMI - body mass index, education, blood lipids

## Abstract

**Purpose:**

Utilize Mendelian randomization (MR) to examine the impact of leisure sedentary behavior (LSB) on the prevalence of type 2 diabetes mellitus (T2D) and glycemic homeostasis impairment, as well as to identify potential mediating pathways involved in these associations.

**Methods:**

We chose genetic variants linked to LSB from a large genome-wide association study (GWAS) to use as instrumental variables (IVs). Then, we used a two-sample MR study to investigate the link between LSB and T2D and glycemic homeostasis. Multivariate MR (MVMR) and mediation analysis were also used to look at possible mediating paths.

**Results:**

MR analysis showed a genetical link between leisure TV watching and T2D (OR 1.64, 95% CI 1.39-1.93, P< 0.001) and impaired Glycemic Homeostasis, while leisure computer use seemed to protect against T2D prevalence (OR 0.65, 95% CI 0.50-0.84, P< 0.001). It was found that leisure TV watching increases the risk of T2D through higher BMI (mediation effect 0.23, 95% CI 0.11-0.35, P< 0.001), higher triglycerides (mediation effect 0.07, 95% CI 0.04-0.11, P< 0.001), and less education (mediation effect 0.16, 95% CI 0.08-0.24, P< 0.001). Sensitivity and heterogeneity analyses further substantiated the robustness of these findings. Reverse MR analysis did not yield significant results.

**Conclusion:**

This study shows LSB is linked to a higher rate of T2D and impaired glycemic homeostasis through obesity, lipid metabolism disorders, and reduced educational attainment.

## 1 Introduction

The incidence and prevalence of type 2 diabetes (T2D) are on the rise worldwide, with the growth rate expected to double that of the neonatal population in the near future (1). This makes T2D a significant public health concern, necessitating urgent improvements in prevention and treatment strategies (2). Peripheral tissue insulin resistance and islet β-cell dysfunction caused by hyperglycemia, relative insulin deficiency, and impaired Glycemic Homeostasis can result in substantial organ damage throughout the body, increasing the risk of cardiovascular and cerebrovascular diseases (3). These conditions impose considerable physical, mental, and economic burdens on affected individuals (2).

With advancing technology, sedentary behavior is becoming increasingly prevalent (4). This encompasses various low-energy expenditure physical activities (typically ≤1.5 METS (5), or metabolic equivalents) performed while awake. Leisure sedentary behavior (LSB) encompasses activities such as using a computer, watching TV, driving, and playing video games. Previous observational studies have identified associations between sedentary behavior and numerous adverse health effects, including obesity (6), T2D (7), cardiovascular disease (8), and certain cancers (9). However, Establishing a causal relationship between sedentary behavior and T2D remains difficult due to the confounding variables, measurement errors, and potential for reverse causality inherent in observational studies (10).

Mendelian randomization (MR), an epidemiological statistical approach, leverages genome-wide association study (GWAS) data to explore causal relationships between exposure factors and diseases at the genetic level (11). This method utilizes genetic variation as an instrumental variable (IV), providing a distinct advantage over traditional observational studies that are often subject to confounding bias. Due to the random assignment of genetic variants during early embryonic development, MR studies are less prone to errors from acquired development and other confounding factors (12).

Herein, to avoid the limitations of traditional observational studies, we used large-scale GWAS data to identify IVs suitable for MR analysis. This allowed us to This allowed us to assess bidirectional causality between LSB and T2D and impaired glucose homeostasis at the genetic level. In addition, we focused on exploring the mechanisms of action of potential mediators influencing the association through two-step MR and mediation analyses. Our study provides constructive suggestions for policy makers and health organizations to guide specific populations more scientifically and accurately to avoid serious health risks associated with sedentary behaviors and to provide preventive intervention strategies.

## 2 Materials and methods

### 2.1 Overview of the study design

**Figure 1:**
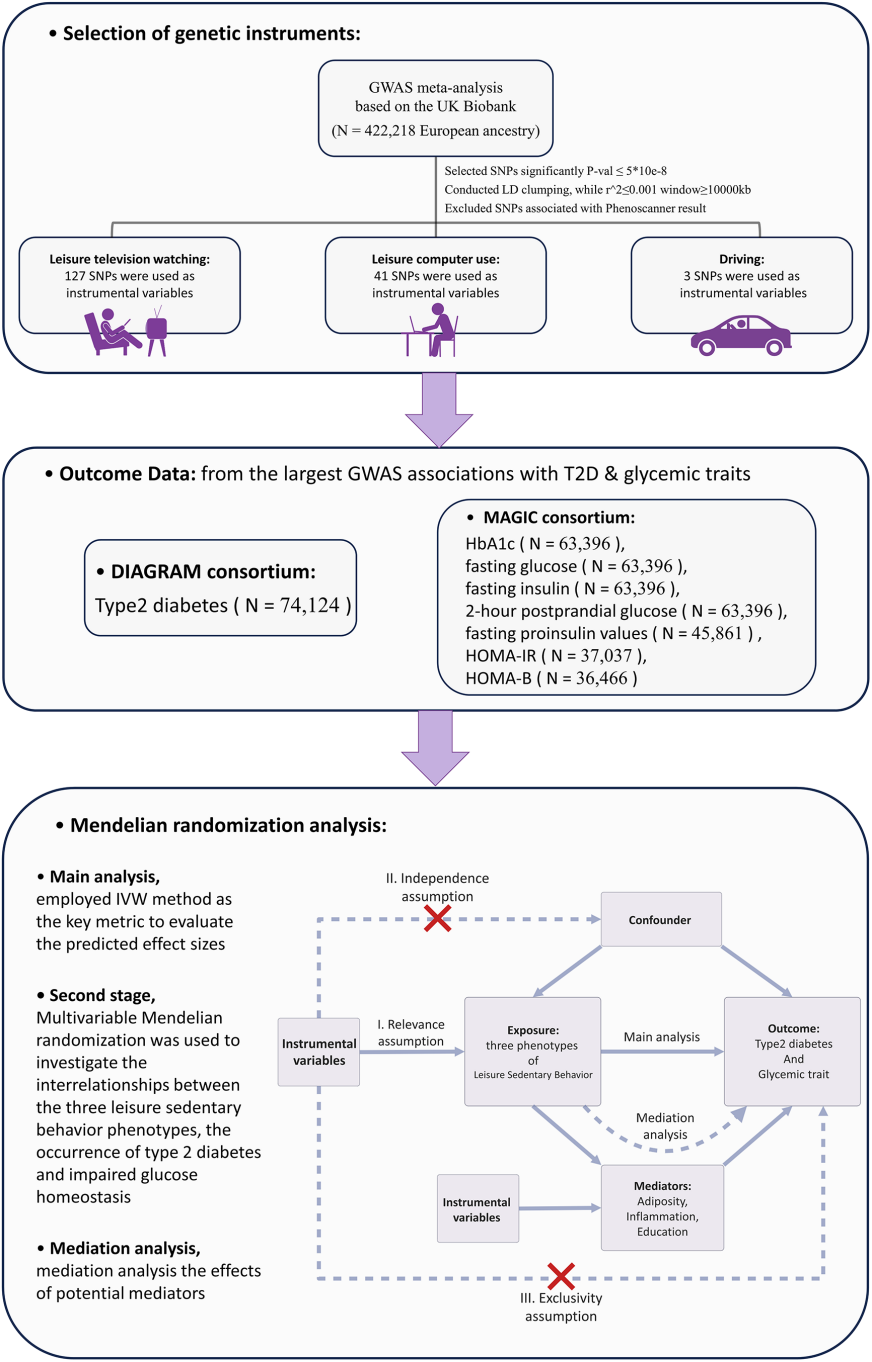
Overview of the Research Methodology. GWAS, Genome-wide association study; IVW, Inverse variance weighted; HbA1c, Glycated hemoglobin; HOMA-IR, Homeostatic Model Assessment for Insulin Resistance; HOMA-B, Homeostatic Model Assessment for Beta-cell Function; SNP, Single nucleotide polymorphism; LD, linkage disequilibrium.

To ensure the integrity of the MR analysis, this study adhered to the STROBE-MR guidelines framework (13). The exposure factors were selected from the largest available public GWAS database, from which SNPs representing LSB were screened as IVs for genetic prediction. The outcome factors were chosen from the largest non-overlapping GWAS data on T2D and impaired glycemic homeostasis. In the first stage, two-sample MR was employed to derive significant causal relationships. In the second stage, utilizing multivariate MR, we investigated the interrelationships among three LSB phenotypes, the occurrence of T2D, and impaired glycemic homeostasis. In the third stage, the potential mediating pathways between LSB and T2D and glycemic homeostasis impairment were examined, along with their genetically predicted effects. The flowchart of the overall study design is shown in **Figure 1**. The STROBE-MR guidelines2 checklist is presented in **Supplementary Table 1**.

### 2.2 Data sources and instrument selection

#### 2.2.1 Selection of genetic IVs for exposure factors

The exposure factor selected for this study was LSB. For this exposure indicator, a GWAS meta-analysis (14) based on the UK Biobank, the most recent and largest sample size available, was chosen. A questionnaire was administered to 422,218 respondents of European ancestry to obtain data on LSB. Three categories were investigated: leisure television watching, leisure computer use, and driving. DNA samples were obtained from whole blood, using the UK Biobank Axiom Array, and genotyping of genetic variant loci. For the three LSB factors mentioned above, SNPs were further screened to obtain IVs. First, all SNPs had to meet a genomic significance P-value of less than 5*10^-8^, which is a widely accepted threshold for genome-wide significance in GWAS (15). Second, for all SNPs in linkage disequilibrium (LD), we set a limit of *R* ^2^ of 0.001 (16), clumping window greater than 10 MB, and removed the palindromic SNPs. The (R^2 = 0.001) threshold ensures that the selected SNPs provide largely independent evidence. Third, each SNP was searched by using the Phenoscanner GWAS database (17), (http://www.phenoscanner.medschl.cam.ac.uk/), and the SNPs among them that might cause potentially pleiotropic were removed. Those removed SNPs are listed in **Supplementary Table 2**.

#### 2.2.2 Selection of outcome data sources

The outcome factors selected for this study included T2D and impaired glycemic homeostasis indicators: glycated hemoglobin A1c (HbA1c), fasting glucose, fasting insulin, fasting proinsulin values, 2-hour postprandial glucose, HOMA-IR, and HOMA-B. For each of these indicators, we opted for the latest GWAS that offers the greatest sample size currently accessible. The final GWAS included in this study were as follows: summary data for T2D from the DIAGRAM (18) study published in 2018 (which can be obtained from http://diagram-consortium.org/downloads.html); summary data for HbA1c, fasting glucose, fasting insulin, 2-hour postprandial glucose (19), fasting proinsulin values (20), HOMA-IR, and HOMA-B (21) from the MAGIC Investigators website.

Relevant articles and data characteristics for the aforementioned study are detailed in **Table 1**.

**Table 1:** Detailed information regarding utilized studies.

Exposure, outcome, or Mediator factors	Definition	Unit	Participants included in the analysis	PMID
Exposure factors				
Leisure television watching	Personally reported questionnaire: “In a typical DAY, how many hours do you spend watching TV?”	hours per day	422,218 European-descent individuals	32317632
Leisure computer use	Personally reported questionnaire: “In a typical DAY, how many hours do you spend using the computer? (Do not include using a computer at work)”	hours per day	422,218 European-descent individuals	32317632
Driving	Personally reported questionnaire: “In a typical DAY, how many hours do you spend driving?”	hours per day	422,218 European-descent individuals	32317632
Mediator factors				
BMI	Body mass divided by the square of the body height	SD (~4.8kg/m^2^)	681,275 European-descent individuals	30124842
Waist circumference	–	SD (cm)	224,459 European-descent individuals	25673412
Hip circumference	–	SD (cm)	224,459 European-descent individuals	25673412
Waist-to-hip ratio	Waist Circumference/Hip Circumference	/	224,459 European-descent individuals	25673412
Body fat Precent	–	%	100,716 European-descent individuals	26833246
Triglyceride	Triglycerides	mmol/L	441,016 European-descent individuals	32203549
CRP	C-reactive protein	mg/L	204,402 European-descent individuals	30388399
Educational attainment	Number of years of schooling completed	years	Over a million European-descent individuals	30038396
Outcome factors				
Type 2 diabetes	binary case-control phenotype of cases with type 2 diabetes mellitus, as defined by a physician diagnosis, Hba1C >=6.5%, or fasting glucose >=126 mg/dl, and controls without evidence of type 2 diabetes	Log odds	74,124 T2D cases and 824,006 controls of European ancestry	30297969
Fasting insulin	–	p mol/L	151,013 European-descent individuals	34059833
Fasting glucose	fasting glucose levels measured in mM among individuals without type 2 diabetes mellitus (not under anti-diabetic medications or insulin, no physician diagnosis of diabetes, fasting glucose levels >7mM) in the MAGIC Consortium	mmol/L	200,622 European-descent individuals	34059833
HbA1c	Glycated Hemoglobin A1c	1%	146,806 European-descent individuals	34059833
Fasting proinsulin values	Proinsulin, a precursor to insulin produced in pancreatic beta cells, may have elevated levels relative to insulin in individuals with prediabetes and type 2 diabetes due to increased demand for beta cells for insulin release.	p mol/L	45,861 European-descent individuals	36693378
2-hour postprandial blood glucose	The blood glucose level taken 2 hours after a meal.	mmol/L	63,396 European-descent individuals	34059833
HOMA-IR	the surrogate estimates of insulin resistance (HOMA-IR) derived from fasting variables by homeostasis model assessment	/	37,037 European-descent individuals	20081858
HOMA-B	the surrogate estimates of β-cell function (HOMA-B) derived from fasting variables by homeostasis model assessment	/	36,466 European-descent individuals	20081858

SD, Standard deviation; HbA1c, Glycated hemoglobin; HOMA-IR, Homeostatic Model Assessment for Insulin Resistance; HOMA-B, Homeostatic Model Assessment for Beta-cell Function.

### 2.3 Statistical analysis

In our two-sample MR analysis, we used the Inverse Variance Weighted (IVW) method as the key metric to evaluate the predicted effect sizes of the causal association between genetically determined LSB and both T2D and impaired glycemic homeostasis. The IVW method has been widely used in TSMR studies, and its validity and accuracy exceed those of other MR analysis methods (22). However, if the study includes invalid IVs in the SNPs, they may affect the outcome through pathways other than exposure factors, leading to significant errors in the IVW method results (23). Compared to the IVW (fixed effects), the IVW (random effects) produce the same beta, but with slightly larger standard errors. IVW (random effects) reduces the bias of results due to the presence of unavoidable heterogeneity, and given the potential heterogeneity between the estimates of different IVs, we chose IVW (random effects) as the main analytical outcome (24). To ensure the reliability of the MR analysis results, we employed a combination of MR-Egger (24), weighted median (25), simple median, and MR-RAPS methods to assess the results jointly. When more than 50% of the included IVs are valid, the weighted median and simple median method results can be considered reliable (24). When the Instrument Strength Independent of Direct Effects (InSIDE) assumption holds, which means that horizontal pleiotropy arises not only due to a single confounder but through multiple confounders and vice versa, the MR-Egger regression method can correct for the effects of genetic predisposition confounding and provide more accurate estimates of causality (24). MR-RAPS (Mendelian Randomization Robust Adjusted Profile Score), which adjusts for genetic predisposition confounding when estimating the result of a cause between the exposure variable and outcome variable, provides stronger robustness to heterogeneity and weak IVs with genetic predisposition confounding.

A reverse MR analysis was performed to assess potential reverse causal effects. In the second stage of Multivariate Mendelian randomization (MVMR) analysis, the independent effect of each LSB phenotype on the causal relationship between T2D and impaired glycemic homeostasis was assessed. The IVW method was similarly employed as the primary outcome for genetic predictive effects in MVMR analysis.

### 2.4 Mediation analysis

Two-sample MR analysis revealed causal associations of genetically predicted LSB with the risk of developing T2D and impaired Glycemic Homeostasis. To investigate potential mediating pathways in the TSMR results, two-step MR and mediation analysis were employed to examine potential factors that play a mediating role in the development of T2D and impaired Glycemic homeostasis. From previous studies, obesity and body size-related factors (BMI (26), body fat percentage (27), waist circumference, hip circumference, waist-hip ratio (28), triglyceride (29)), systemic inflammation level (CRP (30)), and educational attainment (31) were found to be associated with sedentary behavior and T2D onset. Consequently, these factors were incorporated as potential mediators in this stage of the study. The first step investigated the causal association between genetically predicted LSB and potential mediators, using IVW as the primary method (23) to estimate the predicted effect values of LSB for each mediator. The outcomes were presented as correlation coefficients (β) along with their corresponding 95% confidence intervals. For mediators extracted from the same GWAS or database, we used Bonferroni-corrected P-values to assess whether the results were statistically significant, with a corrected p-value of 0.017 for waist circumference, hip circumference, and waist-to-hip ratio. The second step employed MVMR analysis to evaluate the influence of risk factors for T2D (i.e., results with significance in the first step) on the association with the development of T2D after correcting for the effect of LSB. Considering that including too many variables in the study may introduce a more serious problem of covariance, multivariate LASSO regression was used to help screen out unnecessary exposure factors (32, 33).In the third step, based on the above analysis, the predicted values of the respective mediating effects of each risk factor on T2D were obtained using the coefficient product method (34) to assess the proportion of mediating effects of each mediator on T2D.

### 2.5 Sensitivity analysis

MR studies rely on three assumptions (10): 1) genetic IVs selected to represent exposure factors must be strongly correlated with exposure factors; 2) these genetic IVs cannot be correlated with any other confounding factors; 3) these genetic IVs can only affect the outcome event by acting on the exposure factor and cannot be directly correlated with the outcome event. Violation of any of these three assumptions would result in significant errors in the MR study.

For each SNP used as an IV in this study, the strength of the effect was assessed using the F statistic (35), calculated as $F\;statistic=\frac{N-k-1}{k}\left(\frac{R^{2}}{1-R^{2}}\right)$ (when there were less than 5 SNPs: $R_{i}^{2}=2*\beta_{i}^{2}*EAF*1-EAF$ , when there were more than 10 SNPs: $R_{i}^{2}=\frac{2*EAF_{i}*1-EAF_{i}*\beta_{i}^{2}}{2*EAF_{i}*1-EAF_{i}*\beta_{i}^{2}+2*N*EAF_{i}*1-EAF_{i}*se_{i}^{2}}$ ) (36), where EAF is the effect allele frequency, *β_i_* is the estimated genetic effect on LSB, *se_i_* is the standard error of the estimated genetic effect of the exposure, N denotes the sample size of the exposure group, and k signifies the total number of IVs incorporated. An F statistic ≥ 10 is generally considered to indicate that the results are less influenced by weak IVs (37).

In addition, various sensitivity analyses were conducted in this study to examine potential violations of the three main MR assumptions (10) and potential errors in the IVW method results. Cochran’s Q test and the I² statistic were employed to assess heterogeneity and gauge the consistency of the IVs in estimating causal effects. A P-value of 0.05 or lower indicated the existence of pleiotropy (38). When heterogeneity was present, the IVW method served as the primary outcome of the MR analysis. Sensitivity was evaluated using MRPRESSO and leave-one-out test analyses, examining the effect of each SNP on the association with the outcome to ensure the results were free from statistical horizontal pleiotropy (39). Pleiotropy presence was assessed using the intercept obtained from MR-Egger regression (40), with P< 0.05 indicating statistically significant pleiotropy. In this study, we integrated and formatted the GWAS summary data for exposure and outcome variables. By sampling one million unique variants in the formatted data, we estimated a range of interference parameters. Ultimately, we employed the formatted data, estimated interference parameters, and a set of variants adjusted for LD to fit the CAUSE model, which enabled us to evaluate the likelihood of causal and sharing effects (41).

All data analyses in this study utilized R 4.2.2 software and several associated R packages, including TwoSampleMR (ver.0.56) (42), MRPRESSO (ver.1.0) (39), Mendelian Randomization (ver.0.7), and CAUSE (version 1.2.0) (41).

## 3 Result

### 3.1 Genetic IVs selection and F-statistics

We utilized the GWAS associated with LSB from the UK Biobank. Following the exclusion of SNPs that failed to meet the genome-wide correlation threshold (5*10^-8^) and the removal of SNPs in LD with higher P-values, we subsequently screened and eliminated SNPs that might potentially cause confounding bias. Ultimately, three phenotypic SNPs were obtained as IVs. The GWAS features and related literature used in the study are shown in **Table 1**. The F statistics for all SNPs included and used in this study are presented in **Supplementary Tables 2**, **3**.

### 3.2 Two-sample univariate Mendelian randomization analysis

**Figure 2:**
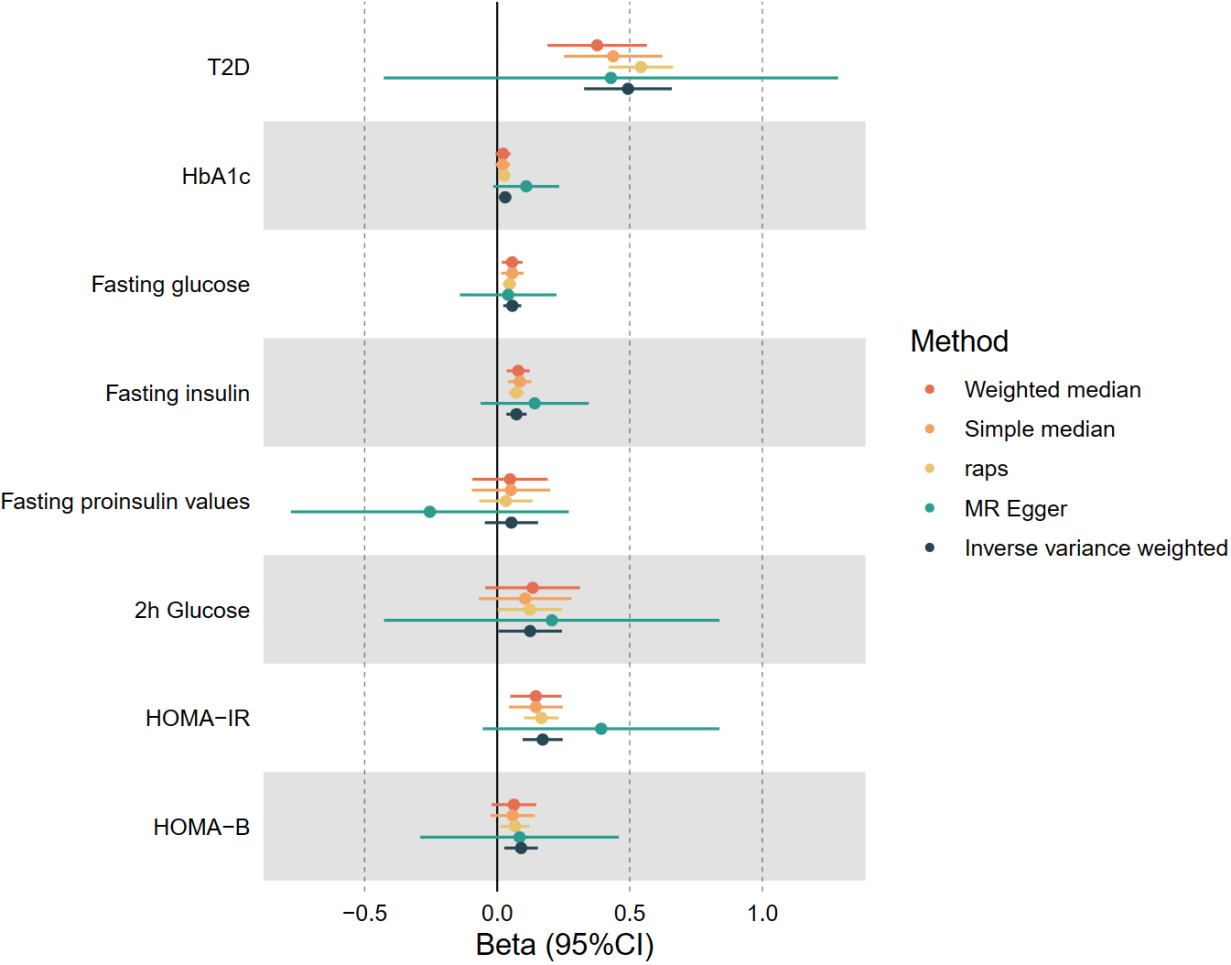
The causal estimates of genetically predicted Leisure Television Watching Behavior with type 2 diabetes and Impaired glycemic homeostasis. T2D, type 2 diabetes, HbA1c, Glycated hemoglobin; HOMA-IR, Homeostatic Model Assessment for Insulin Resistance; HOMA-B, Homeostatic Model Assessment for Beta-cell Function.

In the first stage, the two-sample MR results indicated a significant positive causal relationship between genetically predicted leisure television watching behavior and T2D prevalence, as well as glycemic homeostatic impairment (including HbA1c, fasting glucose, fasting insulin, 2-hour glucose, HOMA-IR, and HOMA-B). After log-transformation to odds ratio (OR) values, each one log odds increase in leisure television watching time was associated with a 64% increase in the prevalence of T2D (OR 1.64, 95% CI 1.39-1.93, P< 0.001), a 3% increase in HbA1c (OR 1.03, 95% CI 1.01-1.05, P< 0.05), a 6% increase in fasting glucose(OR 1.06, 95% CI 1.02-1.10, P< 0.001), an 8% increase in fasting insulin (OR 1.08, 95% CI 1.04-1.12, P< 0.05), a 13% increase in 2h glycemic (OR 1.13, 95% CI 1.00-1.28, P< 0.05), a 19% increase in HOMA-IR (OR 1.19, 95% CI 1.10-1.28, P< 0.001), and a 10% increase in HOMA-B (OR 1.10, 95% CI 1.03-1.17, P< 0.05). The results of the four complementary methods—MR-Egger, simple median, weighted median, and MR-RAPS—remained consistent (as shown in **Figure 2**). The wider confidence intervals for MR-Egger compared to the other methods may be due to its weaker statistical power relative to the IVW method (43). Cochran’s Q statistic in the heterogeneity test ranged from 87.0 to 209.4 (P = 7.76*10^-8^ - 0.53). The MR-Egger intercept analysis in the test of pleiotropy did not exhibit statistically significant directional pleiotropy (P > 0.05). MR-PRESSO showed no impact on the significance of the results after removing potential outlier loci. The leave-one-out sensitivity analysis demonstrated that the overall results’ statistical significance was not substantially impacted by the MR outcomes of other IVs after sequentially removing SNPs. No significant causal association was found between leisure TV watching behavior and fasting insulinogenic values at the genetic level. **Figure 2** depicts a forest plot of the risk association between leisure TV watching behavior and T2D prevalence and impaired Glycemic homeostasis, with different colors representing the results of various study methods.

A significant negative causal relationship was observed between leisure computer use and the prevalence of T2D, with a 35% reduction in T2D prevalence per 1 log odds increase in leisure computer use time after log-transformation to OR (OR 0.65, 95% CI 0.50-0.84, P< 0.001). No notable gene-level associations were identified between leisure computer use and other forms of glycemic homeostasis impairment. The results of the four complementary methods remained consistent. Cochran’s Q statistic equaled 60.0 (P< 0.05) in the test of heterogeneity. MR-PRESSO in the test for pleiotropy did not identify potentially pleiotropic outlier loci. Additionally, no evidence of targeted pleiotropy was found by MR-Egger intercept analysis (P > 0.05).

**Figure 3:**
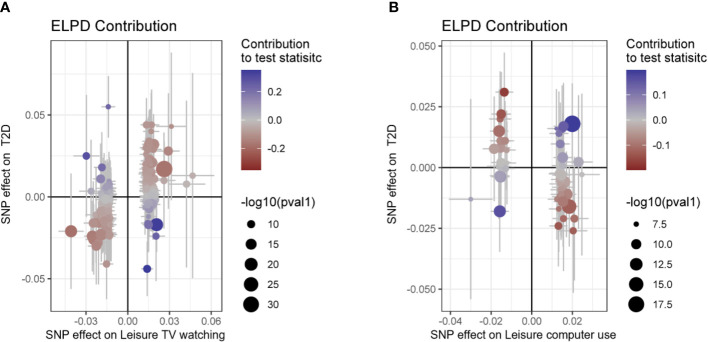
Scatter plot for the CAUSE analysis of the genetically predicted effect of leisure sedentary behavior on type 2 diabetes. **(A)** Scatterplot of CAUSE test statistics for causal association between leisure TV watching behavior and type 2 diabetes. **(B)** Scatterplot of CAUSE test statistics for the causal relationship between leisure computer use behavior and type 2 diabetes. The delta Expected Log Pointwise Posterior Density (ELPD) allows one to evaluate the degree of model fit. The ELPD Contribution diagram depicts visually the contribution of each Single Nucleotide Polymorphism (SNP) to the CAUSE test statistic. The p-values of SNPs are negatively log-transformed, and larger circles represent SNPs with lower p-values, indicating stronger associations between genetic variants and exposure (represented on the x-axis). Those SNPs that contribute more to the causal model are depicted in red, while those that contribute more to the sharing model are depicted in blue (41). T2D, type 2 diabetes; SNP, Single nucleotide polymorphism.

Moreover, CAUSE analyses indicated that the causal model exhibited a superior fit compared to the sharing model (P< 0.001) when examining the relationship between leisure TV watching and T2D prevalence, as illustrated in **Figure 3** and **Tables 2**, **3**. This finding suggests a causal association between the two phenotypes. Nevertheless, no significant causal impact of leisure computer use on T2D (P > 0.05) was detected.

**Table 2:** The CAUSE analysis results for the genetically predicted effect of leisure TV watching and leisure computer use on T2D.

model	Leisure TV watching on T2D			Leisure computer use on T2D		
	gamma	eta	q	gamma	eta	q
sharing	NA	0.76 (0.55, 1.03)	0.59 (0.38, 0.77)	NA	-0.29 (-2.12, 1.86)	0.05 (0, 0.27)
causal	0.58 (0.44, 0.74)	-0.03 (-3.08, 1.99)	0.03 (0, 0.24)	-0.17 (-0.45, 0.09)	0.03 (-1.87, 2.08)	0.04 (0, 0.26)

The statistical metric delta_elpd is used to conduct a comparative analysis between the two models by calculating the difference between the ELPDs of the first and second models. A negative value for delta_elpd indicates that the second model provides a better fit. Eta represents the effect of the sharing component, whereas gamma represents the effect of the causal factor. Moreover, q values represent the proportion of variants that exhibit horizontal pleiotropy correlation (41).

**Table 3:** The CAUSE expected log pointwise posterior density (ELPD) results for the genetically predicted effect of leisure TV watching and leisure computer use on T2D.

model1	model2	Leisure TV watching on T2D				Leisure computer use on T2D			
		delta_elpd	se_delta_elpd	z	p	delta_elpd	se_delta_elpd	z	p
null	sharing	-25.76	5.10	-5.05	2.2E-07	0.24	0.15	1.58	0.94
null	causal	-32.54	6.60	-4.93	4.1E-07	0.30	1.16	0.26	0.60
sharing	causal	-6.79	1.64	-4.13	1.8E-05	0.06	1.02	0.06	0.52

At the genetic level, no significant causal associations were found between driving behavior and T2D and glycemic homeostasis impairment. The results of the TSMR analysis between LSB exposure factors and T2D and Glycemic Homeostasis impairment are presented in **Supplementary Table 4**.

Additionally, we conducted a reverse MR analysis to determine the genetic basis for the association between diabetes, glycemic homeostasis impairment, and sedentary leisure behaviors, but did not obtain statistically significant results. The findings are displayed in **Supplementary Table 5**.

### 3.3 Multivariate Mendelian randomization analysis

In the second stage, considering the potential interaction between LSB, we performed MVMR to assess the direct effects of different LSB on T2D and glycemic homeostatic impairment after accounting for gene-level interactions. After adjusting for the effect of leisure computer use on leisure TV watching behavior, the results for T2D and impairment of glycemic homeostasis remained consistent with univariate TSMR, except that the results for 2-hour postprandial glucose became nonsignificant (P=0.77). After controlling for the effect of driving behavior on leisure TV watching behavior, the results aligned with those of TSMR. Moreover, after correcting for the effect of Leisure watching TV behavior on driving behavior, a significant causal association between driving behavior and 2h postprandial glucose emerged (P< 0.05). Detailed results can be found in **Supplementary Table 7**.

### 3.4 Mediation analysis

In the third stage of the study, the predicted effect values from the first step of genetic prediction of LSB and potential mediation analysis revealed that for each 1 standard deviation (SD) increase in the IVs for genetic prediction of leisure television viewing, the corresponding increase in BMI (effect prediction coefficient β=0.28, 95% CI 0.18-0.39, P<0.001), body fat percentage (effect prediction coefficient β=0.28, 95% CI 0.17-0.40, P<0.001), CRP (effect prediction coefficient β= 0.13, 95% CI 0.04-0.21, P< 0.05), waist-to-hip ratio (effect prediction coefficient β= 0.16, 95% CI 0.08-0.25, P< 0.001), and triglycerides (effect prediction coefficient β= 0.26, 95% CI 0.20-0.32, P< 0.001) was observed. Conversely, the years of education (effect prediction coefficient β= -0.47, 95% CI -0.52- -0.42, P< 0.001) levels decreased. The correlation between LSB and waist and hip circumference was not statistically significant.

For each 1 SD increase in the IV for genetic prediction of leisure computer use behavior, there was a corresponding increase in educational attainment (effect prediction coefficient β=0.54, 95% CI 0.39-0.62, P<0.001). However, no statistically significant causal associations were observed for obesity and inflammation-related indicators. All these results met the threshold range of significant P-values after Bonferroni correction. The detailed results are presented in **Supplementary Table 8**.

In the second step of the MVMR analysis for leisure TV watching behavior and its potential mediators, we found that the LASSO regression screening removed the CRP factor. The results of the remaining mediator analysis demonstrated that, after correcting for the effect of TV watching behavior, the prevalence of T2D increased by 126% (OR 2.26, 95% CI 1.95-2.61, P< 0.001), 33% (OR 1.33, 95% CI 1.18-1.49, P< 0.001), and 64% (OR 1.64, 95% CI 1.31-2.06, P< 0.001) for each 1-unit SD increase in the levels of genetic IVs representing BMI, triglycerides, and waist-to-hip ratio, respectively. The results for body fat percentage (P = 0.25) were not significant.

In a MVMR analysis examining leisure computer use and its potential mediators, we found that the LASSO regression screening removed the years of school factor. Upon completion of the screening and removal process in the initial two stages of the study, BMI, triglycerides, waist-to-hip ratio, and years of schooling emerged as potential factors influencing the prevalence of T2D associated with leisure TV watching behavior.

In the third step, mediation analysis assessed the effect values of genetic prediction for the potential mediators. The mediation analysis results indicated that the proportion of mediating effects of BMI, triglycerides, and educational attainment on the causal relationship between leisure television watching and the occurrence of T2D was 46.57% (95% CI 33.98%-59.16%, P< 0.001), 14.94% (95% CI 11.10%-18.78%, P< 0.001), and 32.30% (95% CI 24.40%-40.19%, P< 0.001), respectively. However, the waist-to-hip ratio did not yield statistically significant results (P = 0.16). Additionally, no significant mediators were identified after screening for leisure computer usage behavior.

Consequently, we concluded that obesity (BMI), abnormal lipid metabolism (triglycerides), and reduced educational attainment serve as significant mediators of the increased prevalence of T2D due to leisure television watching behavior.

**Figure 4:**
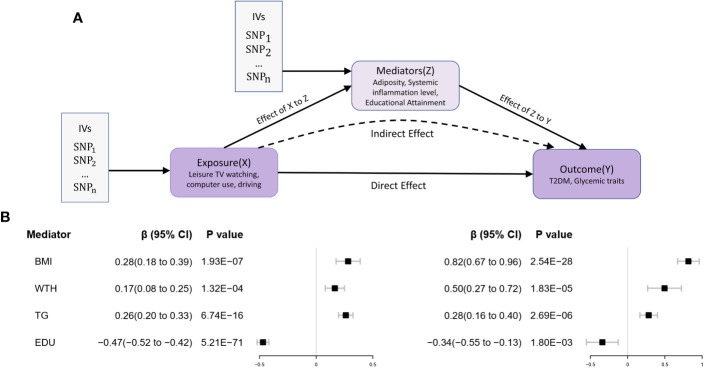
Two-step MR Analysis of the effect of leisure sedentary behavior on type 2 diabetes via potential mediators. **(A)** The two-stage MR analysis framework is designed to systematically assess the causal relationships involved. In the first stage (X to Z), the framework estimates the causal impact of the LSB (X) on potential mediators (Z). The second stage (Z to Y) assesses the causal influence of these mediators on T2D (Y). The “direct effect” denotes the aggregate impact of LSB on T2D risk, while “indirect effects” refer to the effects of LSB on T2D risk that operate through the mediators. Instrumental variables (IVs) are implemented in this analysis. **(B)** Utilizing the MR-IVW method, the genetically predicted effect values for the exposure (X) in relation to the mediator (Z) and the mediator (Z) in association with the outcome (Y) were determined. Error bars represent the 95% confidence intervals (CI). All statistical tests were conducted using a two-sided approach, with a P-value threshold of<0.05 regarded as significant.

As a result, we concluded that obesity (BMI), abnormal lipid metabolism (triglycerides), and reduced educational attainment act as significant mediators in the increased prevalence of T2D associated with leisure television watching behavior. The mediation analysis model diagrams and mediation effect values for the genetic prediction can be found in **Figure 4** and **Table 4**.

**Table 4:** The effect of genetically predicted leisure television watching on T2D via the mediators.

Mediator	Total effect		Direct effect ZY		Direct effect ZY		Mediation effect		Mediated proportion (%)
	β (95% Ci)	se	β (95% Ci)	se	β (95% Ci)	se	β (95% Ci)	P	
BMI	0.49 (0.33 to 0.66)	0.08	0.28 (0.18 to 0.39)	0.05	0.82 (0.67 to 0.96)	0.07	0.23 (0.11 to 0.35)	2.16E-04	46.57% (33.98% to 59.16%)
WTH			0.16 (0.08 to 0.25)	0.04	0.50 (0.27 to 0.72)	0.12	0.08 (-0.03 to 0.19)	0.16	16.57% (4.86% to 28.28%)
TG			0.26 (0.20 to 0.32)	0.03	0.28 (0.16 to 0.40)	0.06	0.07 (0.04 to 0.11)	9.92E-05	14.94% (11.10% to 18.78%)
EDU			-0.47 (-0.52 to -0.42)	0.03	-0.34 (-0.55 to -0.13)	0.11	0.16 (0.08 to 0.24)	4.30E-05	32.30% (24.40% to 40.19%)

“Total effect” refers to the influence of leisure television watching on the incidence of type 2 diabetes (T2D), while “direct effect XZ” refers to the influence of leisure television watching on mediating factors. The “direct effect ZY” refers to the effects of these mediating factors on the risk of T2D, whereas the “mediation effect” refers to the effect of leisure television viewing on the risk of T2D that is mediated by these factors. The total effect, direct effect XZ, and direct effect ZY were calculated using the inverse variance weighted (IVW) technique, whereas the mediation effect was calculated using the coefficient product method. All statistical analyses were two-sided, and a p-value of 0.05 was considered statistically significant.

## 4 Discussion

In this study, we selected reliable IVs representing various phenotypes of LSB for MR analysis. The analysis focused on indicators of T2D onset and impairment of glycemic homeostasis, including glycated hemoglobin A1c (HbA1c), fasting glucose, fasting insulin, 2-hour postprandial glucose, fasting insulin precursors, the surrogate estimates of insulin resistance (HOMA-IR) and β-cell function (HOMA-B) derived from fasting variables by homeostasis model assessment. The objective of this study was to investigate the genetic link between sedentary leisure behavior and the prevalence of T2D and impairment of glycemic homeostasis, as well as to identify potential mediator pathways. Our findings indicated a statistically significant causal association between LSB and T2D, as well as multiple impairments in glycemic homeostasis, which were substantiated using various methods. We subsequently performed mediation analyses to assess potential mediating pathways. The results suggest that LSB contributes to the increased prevalence of T2D and impairment of glycemic homeostasis by causing obesity, lipid metabolism disorders, and decreased educational attainment.

Diabetes and sedentary behavior have been the subject of extensive research. A dose-response meta-analysis investigated the association between sedentary behavior daily and T2D. Their analysis revealed a linear relationship between total sedentary behavior and T2D (P-non-linearity = 0.190), indicating a 5% increase in diabetes risk for each additional hour of sedentary activity per day. A linear association was also observed between television viewing and T2D (non-linearity = 0.948), with an 8% increase in diabetes risk for each extra hour of television viewing per day. In another meta-analysis (44), a linear association between the duration of sedentary behavior and T2D was obtained even after adjusting for physical activity. This revealed a 1% (1.01 [1.00-1.01]) increase in the risk of developing T2D for each additional hour of sedentary time per day. Television viewing was also linearly associated with T2D, with a 9% (1.09 [1.07-1.12]) increase in the risk of developing the disease for each extra hour of daily TV watching. Our findings further support the hypothesis that LSB is associated with an increased threat of T2D.

Current research indicates that insulin resistance resulting from sedentary behavior plays a significant role in poor glycemic control and an increased risk of T2D. Sedentary behavior reduces basal metabolism and the body’s glucose utilization, leading to weight gain. This observation corresponds with our study, which indicates that LSB heightens disease prevalence due to increased BMI. Simultaneously, sedentary behavior, particularly during television viewing, may encourage unconscious consumption of additional food (45), thereby raising average blood glucose levels and stimulating the pancreas to secrete more insulin to lower blood glucose. However, persistent excessive insulin secretion can induce insulin resistance in peripheral cells and a reduction in islet cell function (46). Interrupting extended sitting with standing or walking has been proven in studies to lower postprandial glucose and insulin concentrations (47).

Secondly, extended sedentary periods and diminished contractile activity of skeletal muscles resulting in decreased activity of lipoprotein lipase within the muscles. Lipoprotein lipase is a crucial enzyme regulating lipid metabolism and mediating the uptake of free fatty acids in muscle and adipose tissue (48). Reduced activity of this enzyme is linked to elevated triglyceride levels, decreased HDL cholesterol, and increased glucose levels (49). And the inverse relationship between television viewing duration and educational attainment is evident (50), which aligns with the findings of our study.

Prior research has confirmed that sedentary behavior can lead to metabolic disorders, which can trigger a systemic low-grade chronic inflammatory response. This is characterized by elevated levels of inflammatory factors (51, 52) in the blood, such as leukocytes, Interleukin-6 (IL-6), and C-reactive protein (CRP). These inflammatory factors can further interfere with insulin signaling, reduce insulin sensitivity, and exacerbate poor glycemic control. However, we did not observe a statistically significant correlation between systemic chronic inflammation and the development of T2D owing to sedentary behavior in our study. One possible explanation is that we included only CRP as a proxy for inflammatory indicators, while multiple biological pathways could influence the relationship between sedentary behavior and diabetes. Another consideration is that MR studies are statistical methods at the genetic level and may not fully reveal these complex biological pathways.

Different leisure activities, though they may share similar caloric outputs, can impact health in varying ways due to inherent characteristics of each activity. For instance, television watching, a predominantly passive activity, is often paired with unhealthy snacking, potentially exacerbating the health risks beyond mere sedentary behavior (53). On the other hand, leisure computer use, which might involve cognitive tasks such as learning or strategy games, demands different metabolic and neurological responses (54). Furthermore, while driving also falls into the sedentary category, it is often a necessity rather than a leisure choice. The unique stressors associated with driving, especially in dense traffic situations, present their own health implications (55). Therefore, while caloric expenditure is a critical metric, it is only one facet of a multifaceted narrative, underscoring the need to consider the nuances of each activity when evaluating their health consequences. To further elucidate these associations, future investigations could benefit from a more granular assessment, potentially utilizing real-time monitoring to gain insights into concurrent behaviors during these leisure activities.

There are also studies that contradict our results. For instance, the Norway HUNT Study (56), which followed 28,051 adults for up to 11 years, demonstrated a 17% increased risk of developing T2D in those with over 8 hours per day of daily sedentary time compared to those with less than 4 hours per day of daily sedentary time (HR=1.17, 95% CI). The exact reason for these inconsistent findings is unclear and may be attributable to differences in physical and genetic characteristics, lifestyle behaviors, socioeconomic status, and environmental factors across ethnic groups.

The primary strength of this study lies in the utilization of the latest GWAS data and advanced statistical methods, such as two-sample MR analysis and two-step mediation analysis. These approaches enable low-cost, efficient, and reproducible analyses. We verified genetic correlations and causal associations between LSB, risk of T2D mellitus, and glycemic indicators. Furthermore, we included diabetes high-risk factors and conducted a mediation analysis using MR to determine whether the relationship between LSB and T2D is influenced by additional factors.

We acknowledge several limitations in our study. Firstly, the LSB analyzed in this research (14) were limited to leisure TV watching, computer use, and driving. With the increasing prevalence of technology, including the use of smartphones, has become an integral aspect of daily life (57), potentially impacting health, particularly in relation to T2D and impaired glycemic homeostasis. This study did not account for occupational sedentary behavior or other activities. Furthermore, self-reported data collection on sedentary time (14) introduces the possibility of recall bias.

The outcome study also did not evaluate the severity and complication status of T2D patients (19). Secondly, unidentified or unconsidered potential mediators may exist in the mediated MR analysis. Despite including multiple potential mediators, the complex interplay between sedentary lifestyle and elevated risk of diabetes and glycemic-related indicators remains incompletely explained. Thirdly, our study did not establish statistically significant causal associations between driving behavior and T2D or impaired glycemic homeostasis. While we selected strong instrumental variables (37) with F > 10 effect values, the limited number of SNPs included could result in low explanatory values of the IVs, potentially concealing causal associations. Future research should explore these connections more comprehensively. Finally, the data sample was limited to European-descent individuals. To generalize the findings to other ethnicities or regions, multiethnic-based studies are required.

In conclusion, our study used MR analysis to systematically and robustly demonstrate that LSB at the genetic level causes T2D and impaired glycemic homeostasis through mediating pathways such as increased BMI, increased triglyceride levels, and decreased educational attainment. Sedentary behavior has permeated people’s lives as their lifestyles have changed, and in the current societal context where sedentary behavior is increasingly common, it is important to emphasize and promote diversified preventive measures. These include raising public awareness of the potential risks of sedentary behavior, encouraging positive changes in work and learning environments, and promoting the use of science and technology in health promotion. Future studies should further explore the relationship between these behaviors and their biological effects, search for the most effective intervention strategies, and comprehensively examine the effects of their application in real-world settings. It is hoped that through these comprehensive measures, our findings will further inform research on the relationship between diabetes mellitus and poor lifestyle habits to address the long-term health challenges of sedentary behavior. Therefore, in addition to further exploring the mechanisms of the association between sedentary behavior and the development of T2D, future studies should focus on exploring the appropriate range or harmful threshold of sedentary time to further provide a scientific basis for the prevention of T2D.

## Data availability statement

Publicly available datasets were analyzed in this study. This data can be found here: Leisure Sedentary Behaviors Summary Statistics (https://data.mendeley.com/datasets/mxjj6czsrd/1); Glycemic traits data contributed by MAGIC investigators (https://magicinvestigators.org); T2D data contributed by DIAGRAM consortium (http://diagram-consortium.org/).

## Author contributions

HJ and DL designed the study and analyzed the data. YFL did the literature research and created the tables and plots. HJ, YFL drafted the manuscript. The final manuscript was reviewed by DL. All authors reviewed and approved the final version.
