# Supplementary material for: Role of leisure sedentary behavior on type 2 diabetes and glycemic homeostasis: a Mendelian randomization analysis
Source: Front Endocrinol (Lausanne). 2023 Nov 23;14:1221228. doi: 10.3389/fendo.2023.1221228 (PMC10702218; doi:10.3389/fendo.2023.1221228)

Supplementary Figure 1

Leave-one-out analysis of plots for the relationship of genetically predicted leisure TV watching with T2D

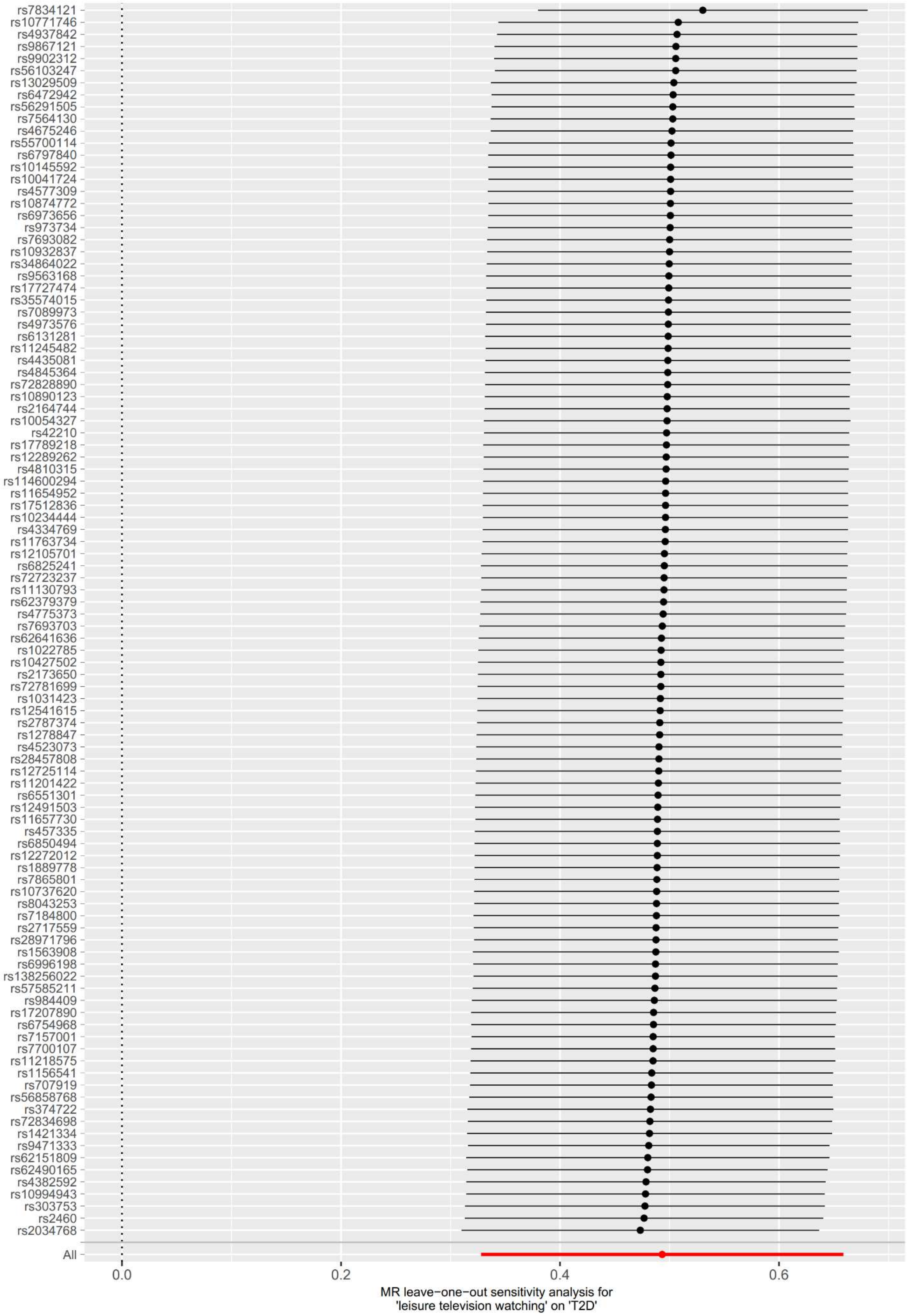

Supplementary Figure 2

Mendelian randomization plots for the relationship of genetically predicted leisure TV watching with HbA1c

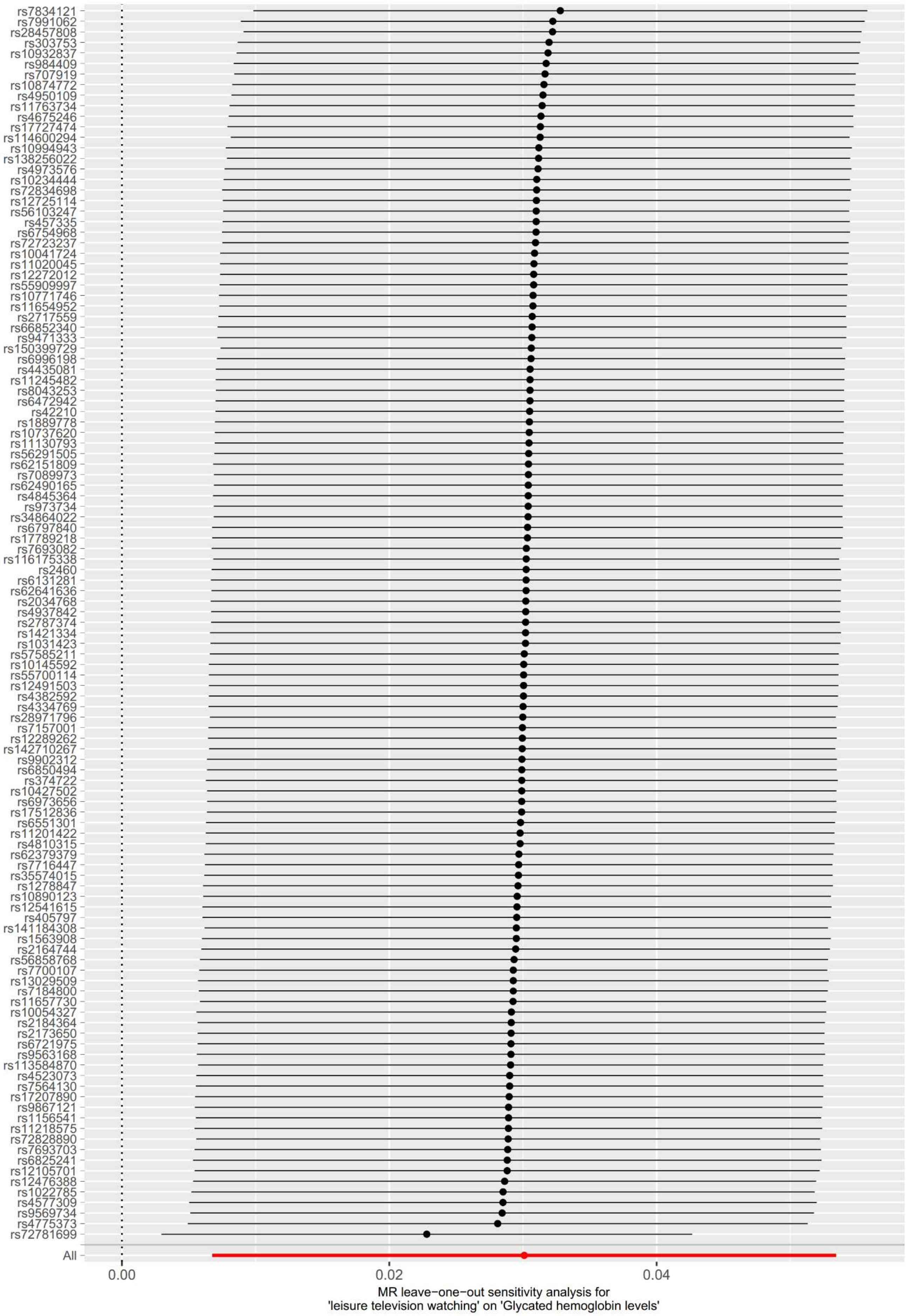

Supplementary Figure 3

Leave-one-out analysis of plots for the relationship of genetically predicted leisure TV watching with Fasting glucose

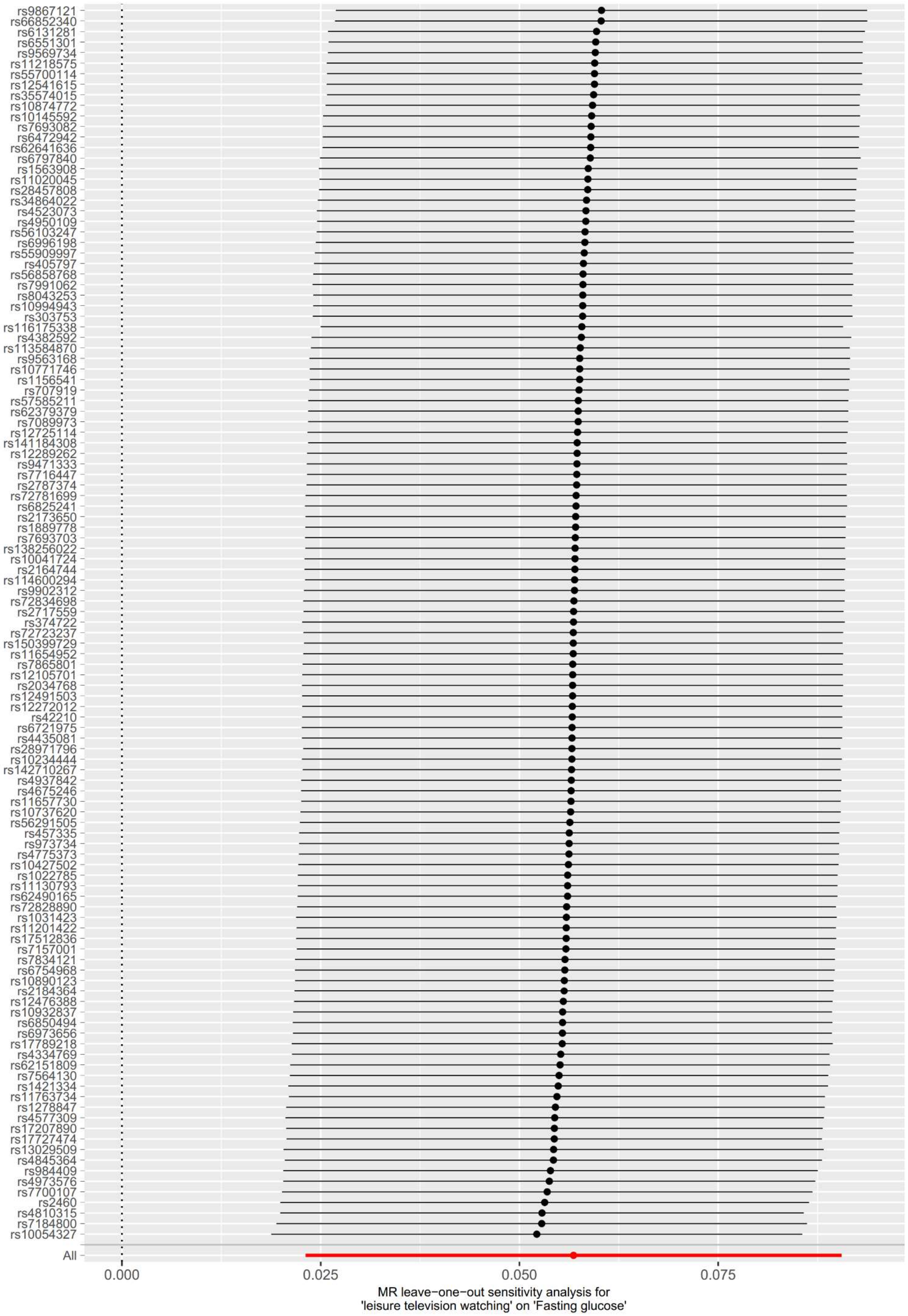

Supplementary Figure 4

Leave-one-out analysis of plots for the relationship of genetically predicted leisure TV watching with Fasting insulin

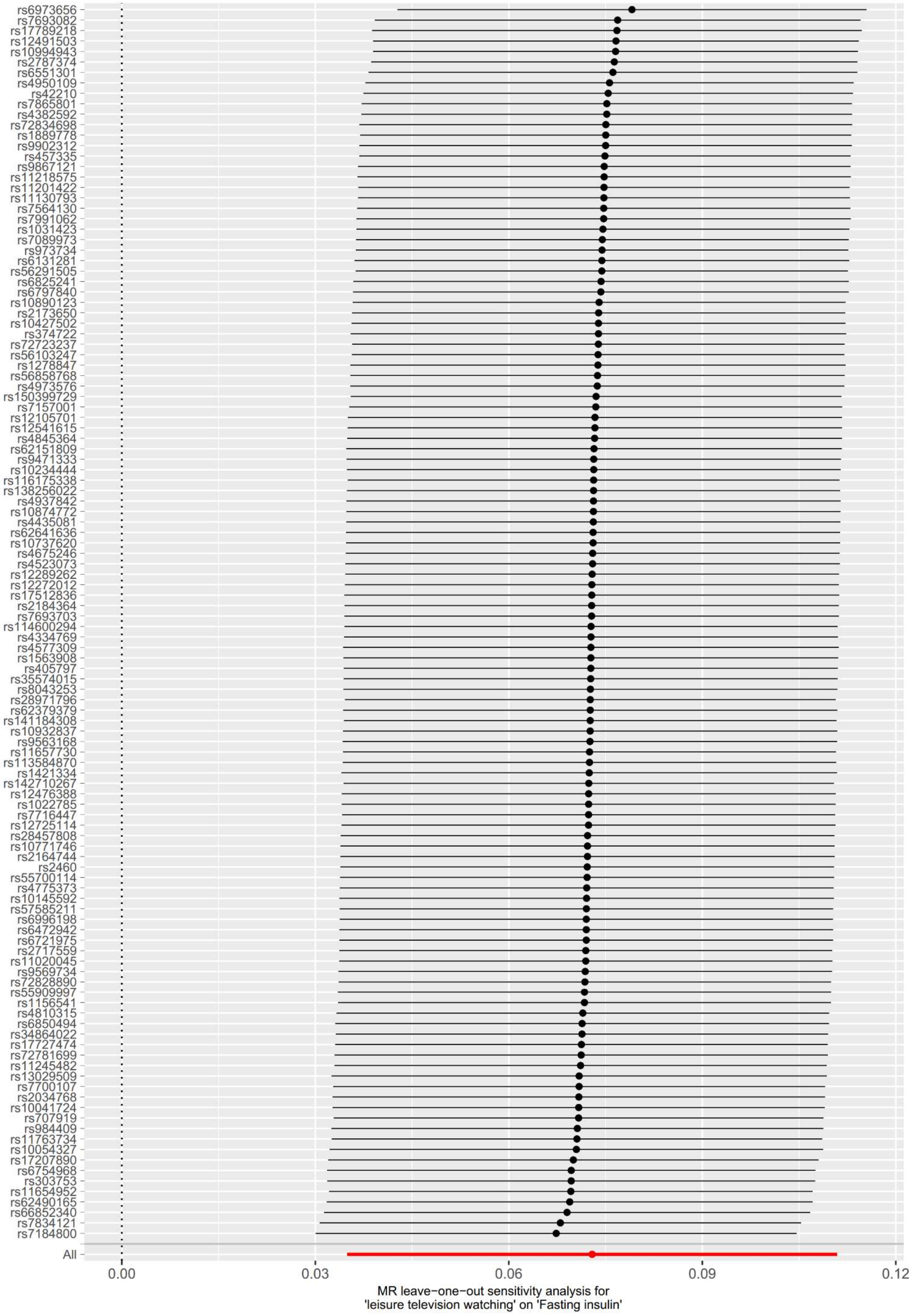

Supplementary Figure 5

Leave-one-out analysis of plots for the relationship of genetically predicted leisure TV watching with Fasting proinsulin value

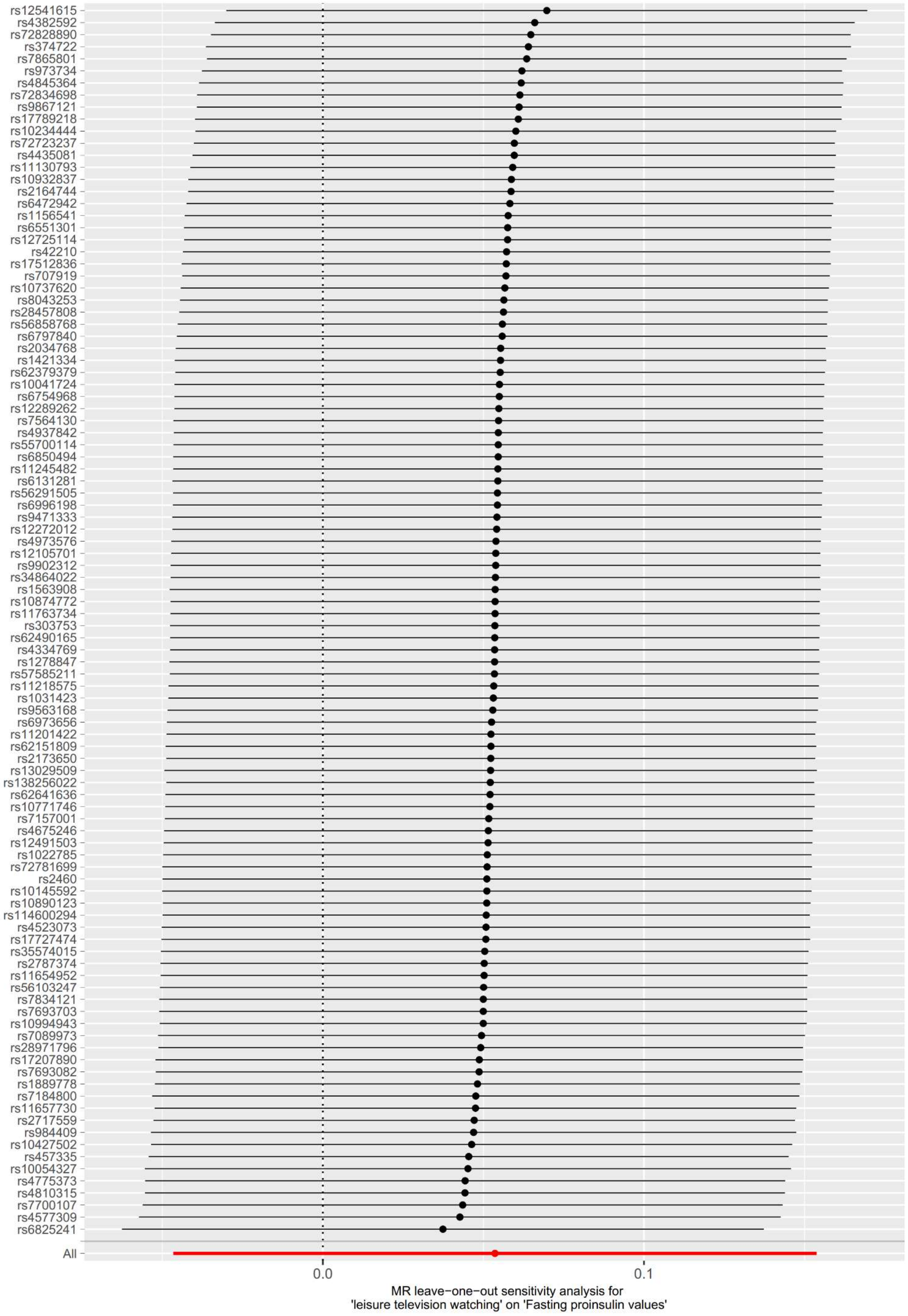

Supplementary Figure 6

Leave-one-out analysis of plots for the relationship of genetically predicted leisure TV watching with 2h Glycemic

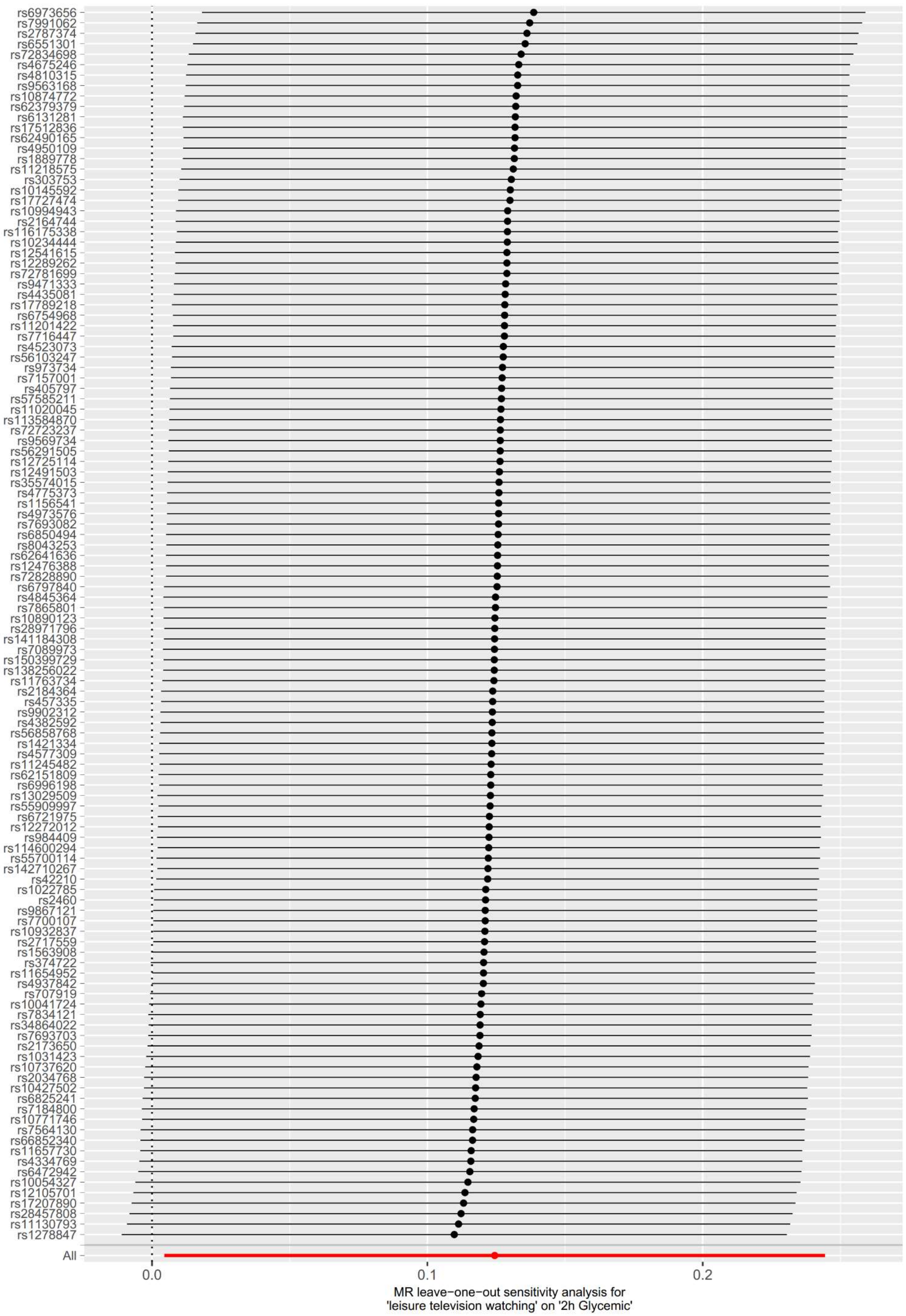

Supplementary Figure 7

Leave-one-out analysis of plots for the relationship of genetically predicted leisure TV watching with HOMA-B

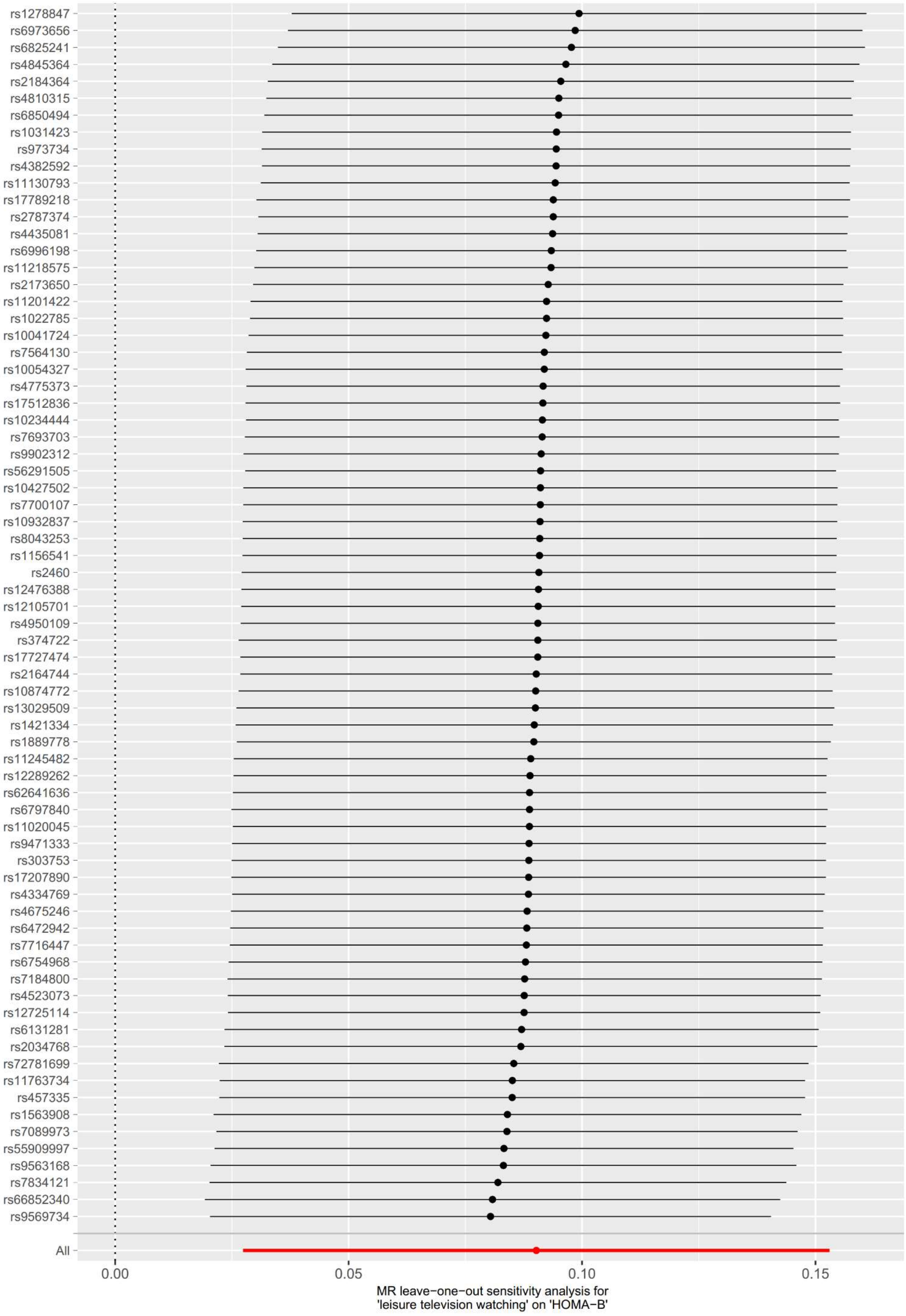

Supplementary Figure 8

Leave-one-out analysis of plots for the relationship of genetically predicted leisure TV watching with HOMA-IR

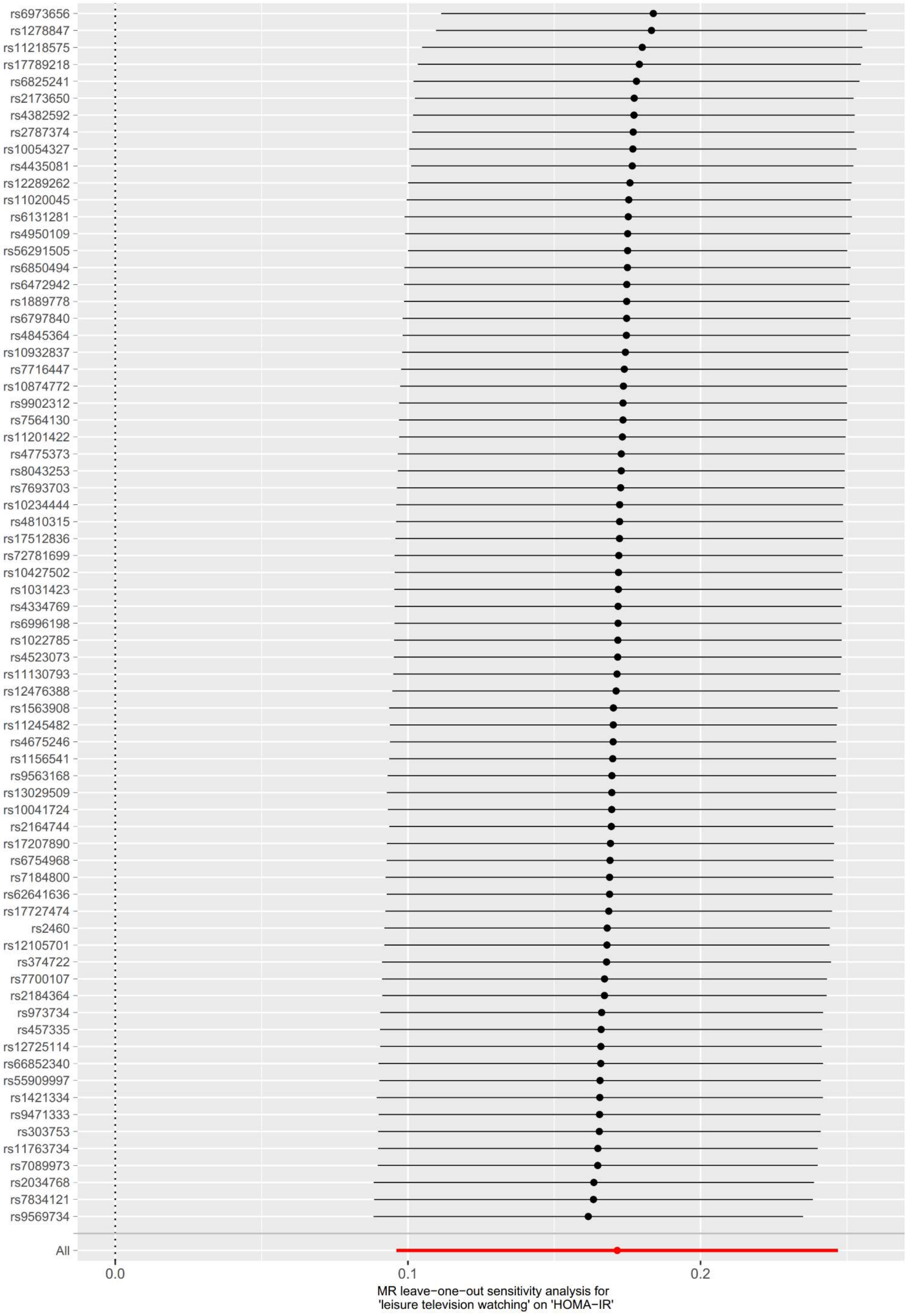

Supplementary Figure 9

Leave-one-out analysis of plots for the relationship of genetically predicted leisure computer use with T2D

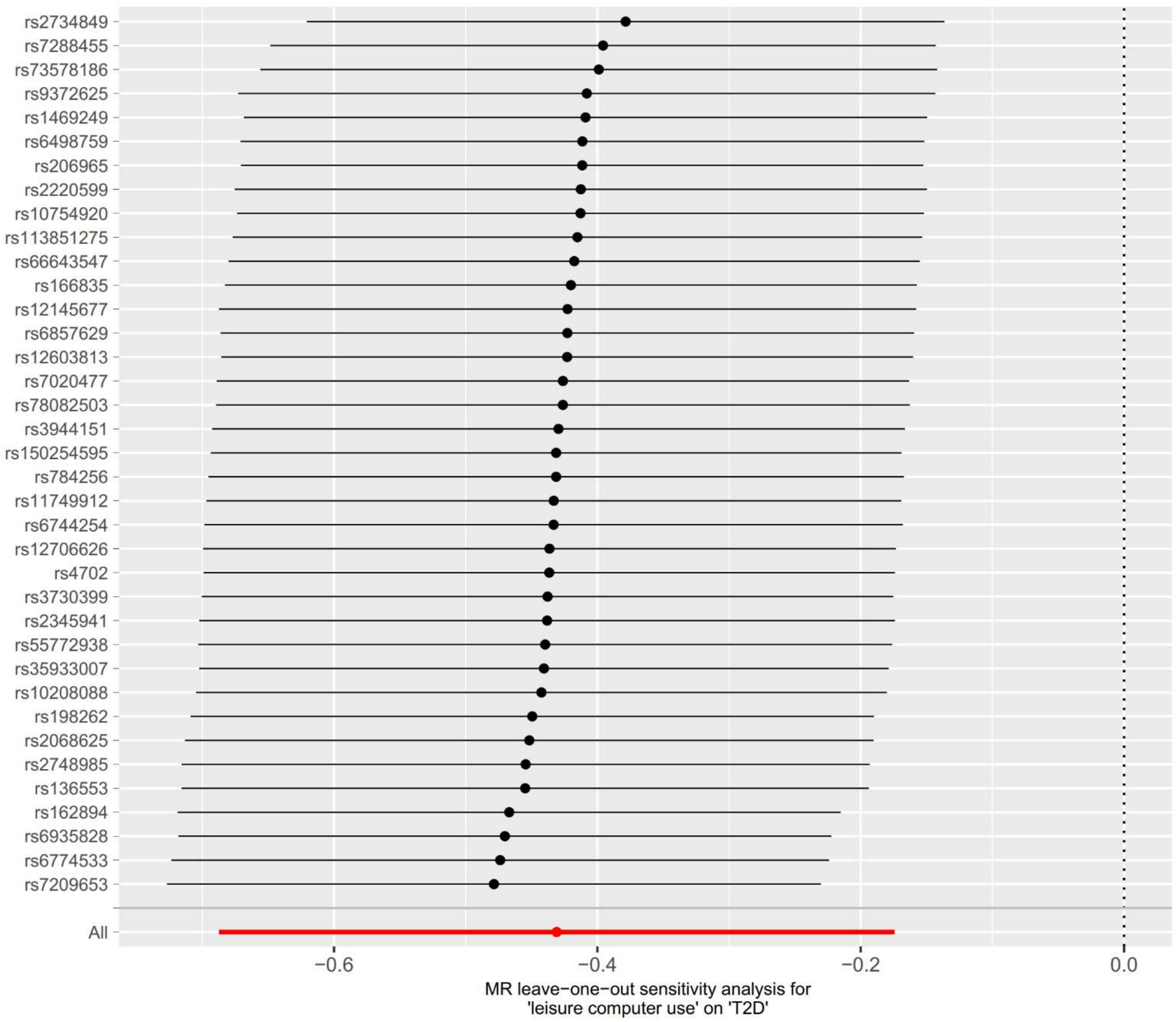

Supplementary Figure 10

Leave-one-out analysis of plots for the relationship of genetically predicted leisure computer use with HbA1C

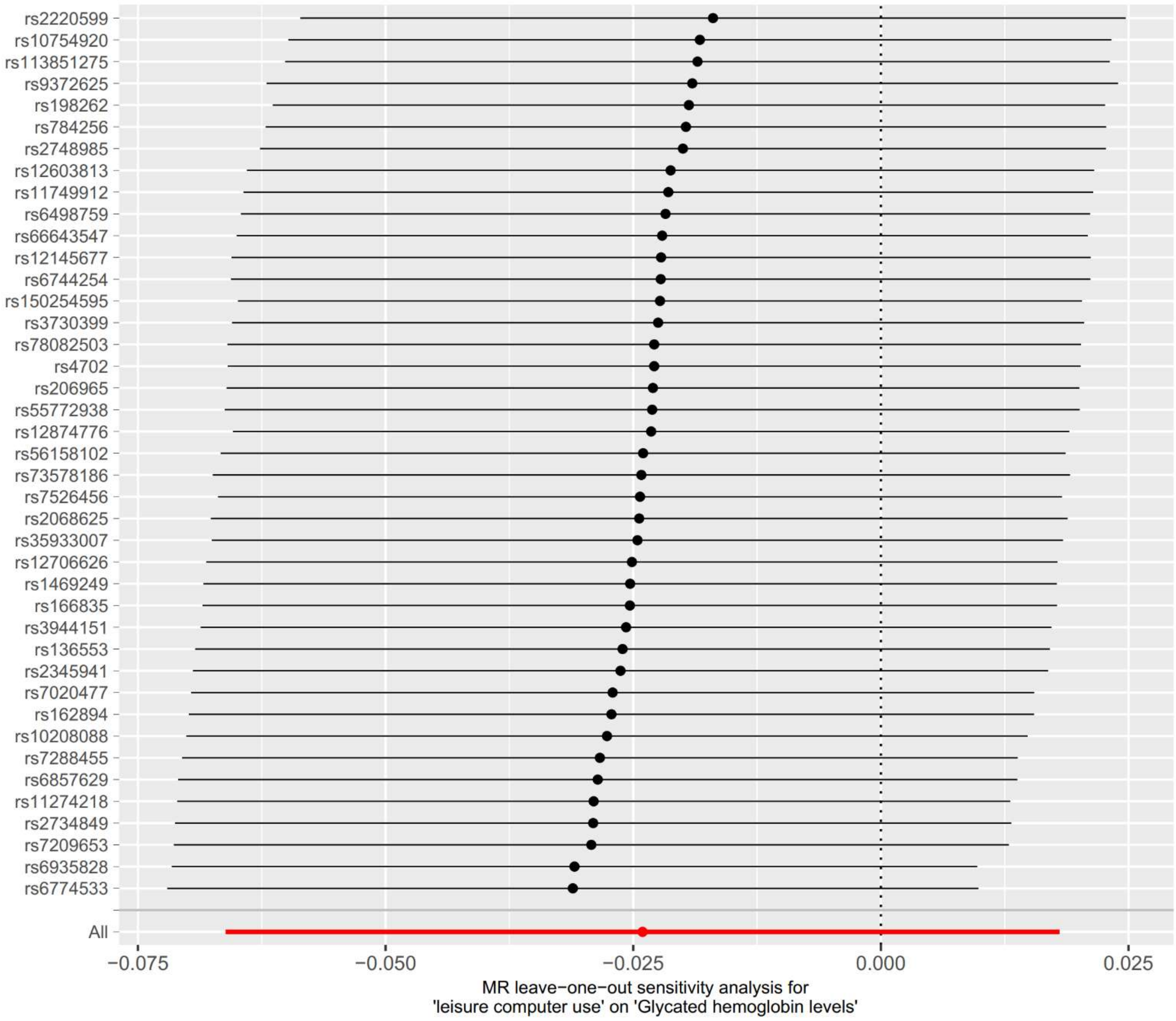

Supplementary Figure 11

Leave-one-out analysis of plots for the relationship of genetically predicted leisure computer use with Fasting Glucose

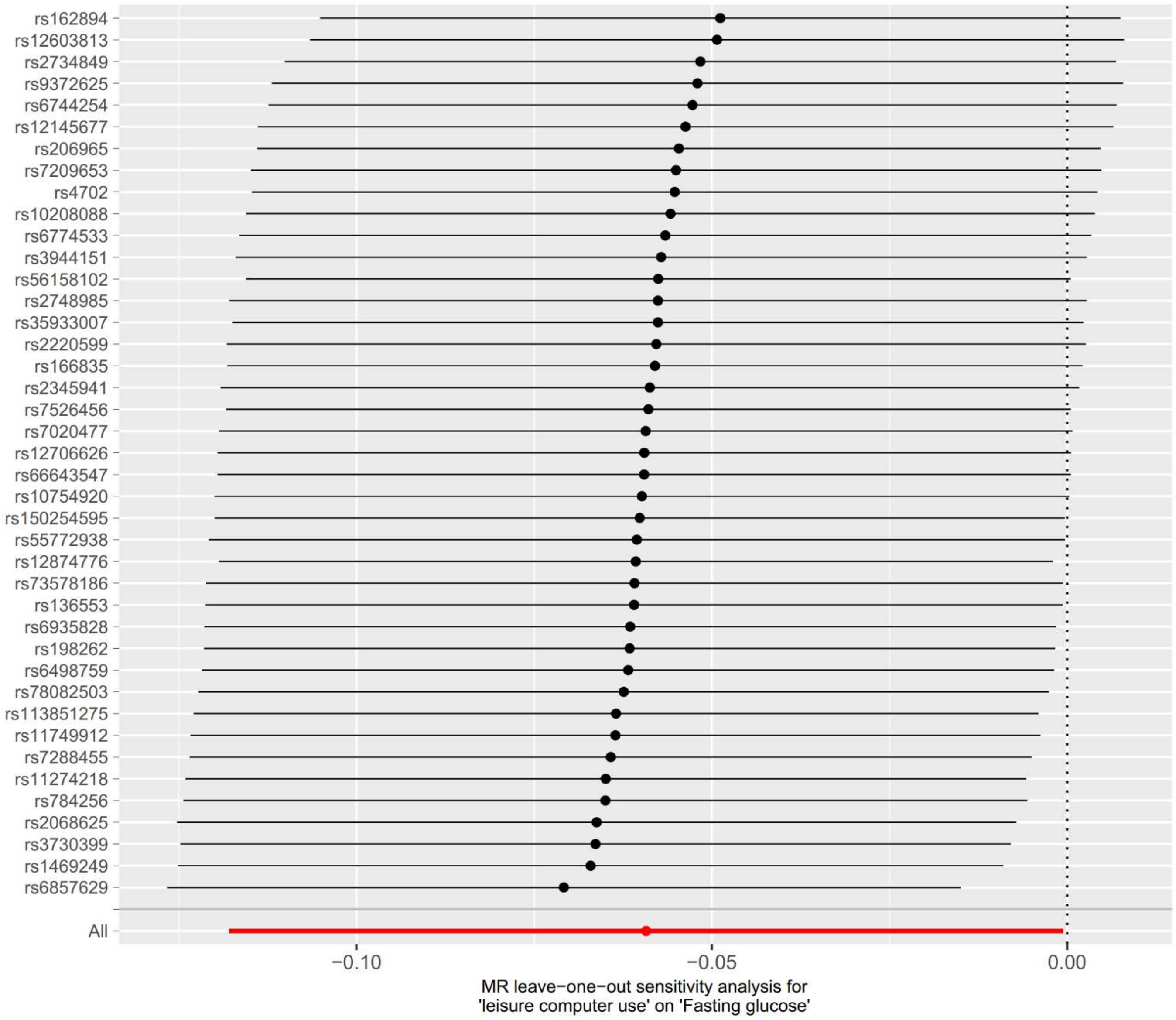

Supplementary Figure 12

Leave-one-out analysis of plots for the relationship of genetically predicted leisure computer use with Fasting insulin

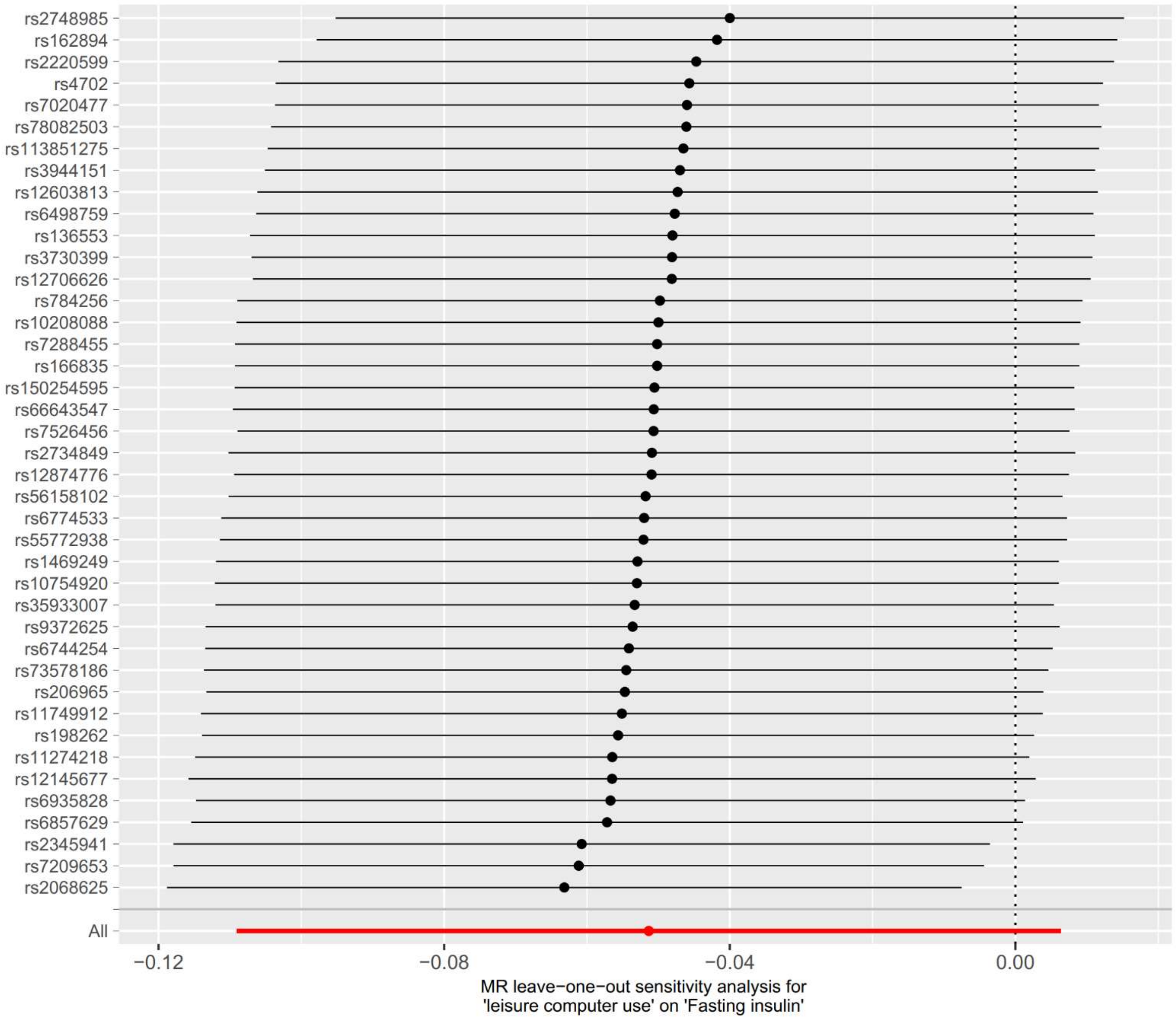

Supplementary Figure 13

Leave-one-out analysis of plots for the relationship of genetically predicted leisure computer use with Fasting proinsulin values

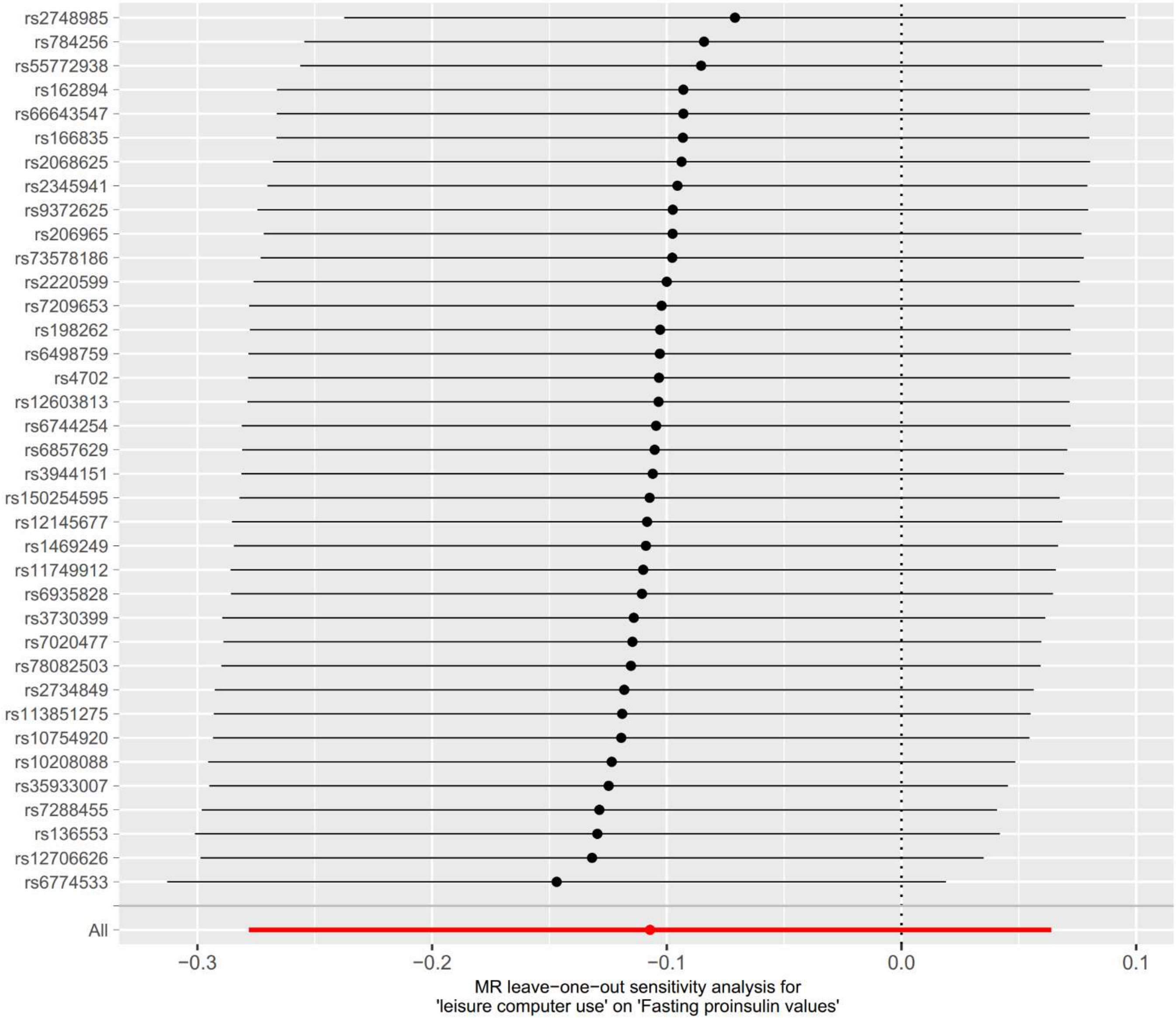

Supplementary Figure 15

Leave-one-out analysis of plots for the relationship of genetically predicted leisure computer use with 2h Glycemic

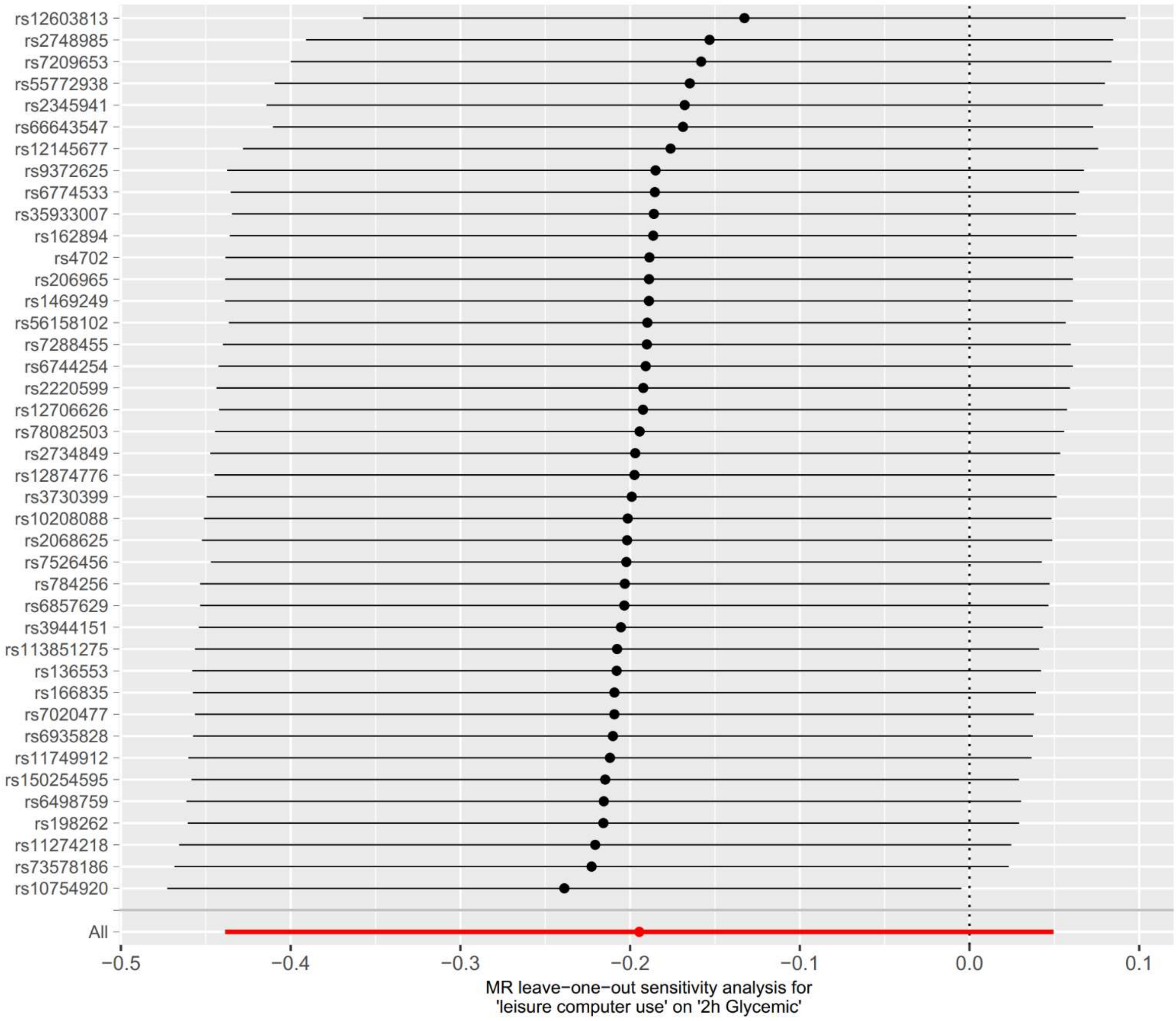

Supplementary Figure 15

Leave-one-out analysis of plots for the relationship of genetically predicted leisure computer use with HOMA-B

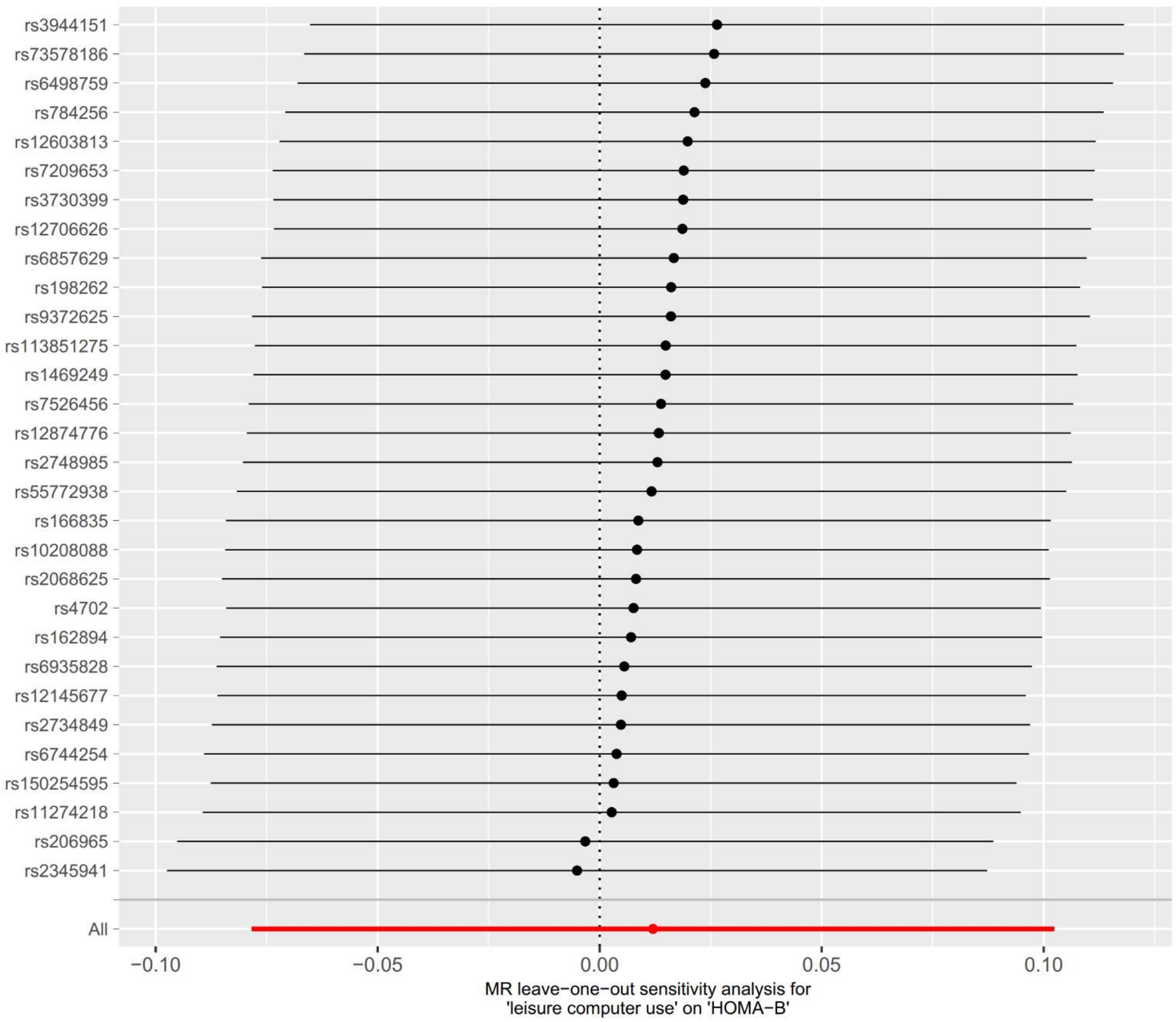

Supplementary Figure 16

Leave-one-out analysis of plots for the relationship of genetically predicted leisure computer use with HOMA-IR

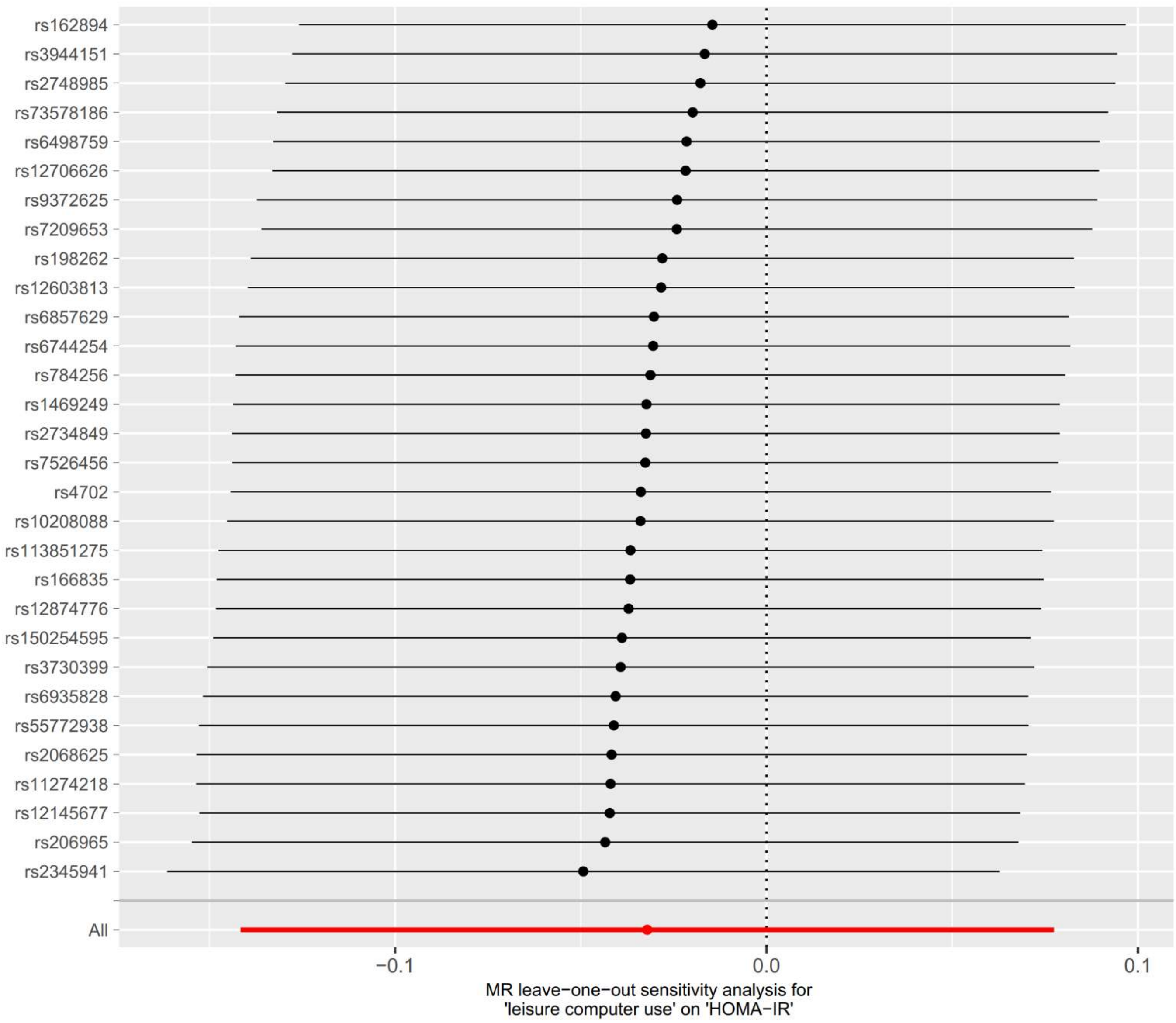

Supplement: Supplementary file 1 [file DataSheet_1.pdf]
